# Transcriptome analysis reveals the host selection fitness mechanisms of the *Rhizoctonia solani* AG1IA pathogen

**DOI:** 10.1038/s41598-017-10804-1

**Published:** 2017-08-31

**Authors:** Yuan Xia, Binghong Fei, Jiayu He, Menglin Zhou, Danhua Zhang, Linxiu Pan, Shuangcheng Li, Yueyang Liang, Lingxia Wang, Jianqing Zhu, Ping Li, Aiping Zheng

**Affiliations:** 1 Rice Research Institute, Sichuan Agricultural University, Chengdu, 611130 China; 2 Key Laboratory of Sichuan Crop Major Diseases, Sichuan Agricultural University, Chengdu, 611130 China; 3 Key Laboratory of Southwest Corp Gene Resource and Genetic Improvement of Ministry of Education, Sichuan Agricultural University, Ya’an, 625014 China

## Abstract

*Rhizoctonia solani* AG1IA is a major generalist pathogen that causes sheath blight. Its genome, which was the first to be sequenced from the *Rhizoctonia* genus, may serve as a model for studying pathogenic mechanisms. To explore the pathogen-host fitness mechanism of sheath-blight fungus, a comprehensive comparative transcriptome ecotype analysis of *R. solani* AG1IA isolated from rice, soybean and corn during infection was performed. Special characteristics in gene expression, gene ontology terms and expression of pathogenesis-associated genes, including genes encoding secreted proteins, candidate effectors, hydrolases, and proteins involved in secondary metabolite production and the MAPK pathway, were revealed. Furthermore, as an important means of pathogenic modulation, diverse alternative splicing of key pathogenic genes in *Rhizoctonia solani* AG1IA during infections of the abovementioned hosts was uncovered for the first time. These important findings of key factors in the pathogenicity of *R. solani* AG1IA ecotypes during infection of various hosts explain host preference and provide novel insights into the pathogenic mechanisms and host-pathogen selection. Furthermore, they provide information on the fitness of *Rhizoctonia*, a severe pathogen with a wide host range.

## Introduction

The soil-borne Basidiomycete fungus, *Rhizoctonia solani Kühn*, is a destructive and widespread plant pathogen that can infect approximately 200 species of crops^1^. According to its anastomosis criterion, ecology and host range, *R. solani* is divided into 14 anastomosis groups (AG1 to AG13 and AGBI)^2^. *R. solani* AG1 consists of six primary intraspecific subgroups (AG1IA, AG1IB, AG1IC, AG1ID, AG1IE and AG1IF)^3, 4^. The AG1IA subgroup is considered the main pathogen that causes sheath blight in rice (*Oryza sativa L*.), maize (*Zea mays L*.) and soybean (*Glycine max*)^5–7^. As one of the most destructive diseases of cultivated rice, rice sheath blight caused by *R. solani* AG1IA can result in yield losses of 50% in Asia^8^. Although resistance to sheath blight has been reported^9^, completely resistant cultivars or immune varieties have not been found^10^. However, most research has been conducted on genus-species and anastomosis groups^11^, and few studies have investigated pathogenicity at the molecular level^12, 13^ or its genetic variability^14–17^.

RNA-sequencing(RNA-Seq) has become a very popular method for elucidating the molecular mechanisms involved in specific biological and disease processes^18^. Using RNA-Seq, much research has been conducted on pathogen-host interactions (PHIs) in major plant diseases, including *Magnaporthe oryzae* ^19^, *Ustilago maydis* ^20^ and *Phytophthora infestans* ^21^. Transcriptomics technology has also played an important role in the study of various pathogens such as *Postia placenta* ^22^, *Colletotrichum* ^23^, *Moniliophthora perniciousa* ^24^ *, Xanthomonas* ^25^, and *Fusarium* ^26^ and their hosts.

Currently, the genomes of *R. solani* AG1IA, AG1IB (isolate 7/3/14), AG3, AG8, and AG2-2IIIB have been sequenced^27–31^ and the genomes of *R. solani* AG1IA, AG1IB, AG3, and AG8 have been compared^32^. In particular, whole-genome sequencing of *R. solani* AG1IA has enabled prediction of pathogenesis-associated genes and effectors, which could lead to novel insights into pathogenic mechanisms^28^. The *R. solani* proteome has increased our understanding of the mechanisms by which *R. solani* adapts to different plant hosts^33^. Furthermore, the transcriptome of *R. solani* AG1IA during early invasion of *Zoysia japonica* roots^34^, and changes in the transcriptome change after infection with *R. solani*, were recently reported^35^. Genome sequencing and annotation have provided a global view of *R. solani* IA genes, and analysis of the transcriptome at six stages of infection has improved our understanding of the mechanism by which *R. solani* AG1 infects rice. Despite considerable advances in our understanding of *R. solani* AG1IA in the past several years, progress in genetic studies and our understanding of pathogenic factors has been limited due to the multinucleate and heterokaryotic nature of *R. solani*, which has hindered our ability to study the host selection fitness mechanisms of sheath-blight fungus. To date, the majority of studies on *R. solani* AG1IA PHIs have focused on comparisons of pathogenicity and biological characteristics in different hosts^36, 37^. Based on this, we provide the first comparative transcriptome analysis of *R. solani* AG1IA gene expression during infection of different hosts and provide novel insights into the host selection fitness mechanisms during infection by sheath-blight fungus. High-throughput sequencing and bioinformatics were used to profile the transcriptomes of three strains of different ecotypes of *R. solani* AG1IA under different infection conditions. Many differentially expressed, pathogenesis-associated genes—including secreted proteins, candidate effectors, carbohydrate-active enzymes (CAZymes), and secondary metabolites involved in the pathogenesis of *R. solani* AG1IA in various hosts were identified. Moreover, alternative splicing of key pathogenesis-related genes in *R. solani* AG1IA during the infection of different hosts was evaluated. By comparing the transcriptomes of *R. solani* AG1IA ecotypes cultivated in the same host and original/non-original hosts to those cultivated on vegetative mycelia, we hope to improve our understanding of the infection mechanism of the sheath blight fungus, *R. solani* AG1IA.

## Results

### Biological characteristics of strains from different hosts

The three strains, rice IA, maize IA, and soybean IA, were randomly isolated from heavily infected rice (*O. sativa L*.), maize (*Z. mays L*.) and soybean (*G. max*) plants in Sichuan province in areas of different altitudes (Xichang, Chengdu, and Yaan, respectively). To explore the biological differences in strains from different host, a comparative analysis including hyphal anastomosis, growing situation and pathogenicity were conducted. Hyphal anastomosis among rice IA, maize IA, and soybean IA showed somatic incompatibility and no fusion of hyphae, and strains from self-pairings were compatible and exhibited no demarcation between paired ecotypes (Supplementary Fig. S1). Cultures of three strains all could anastomose with AG1IA and all ecotypes from rice IA, maize IA, and soybean IA hosts showed identical ribosomal DNA ITS (Internal Transcribed Spacer Identification). We found that some genetic differences still existed after long-term co-evolution of the strains and their host plants.

**Figure 1:**
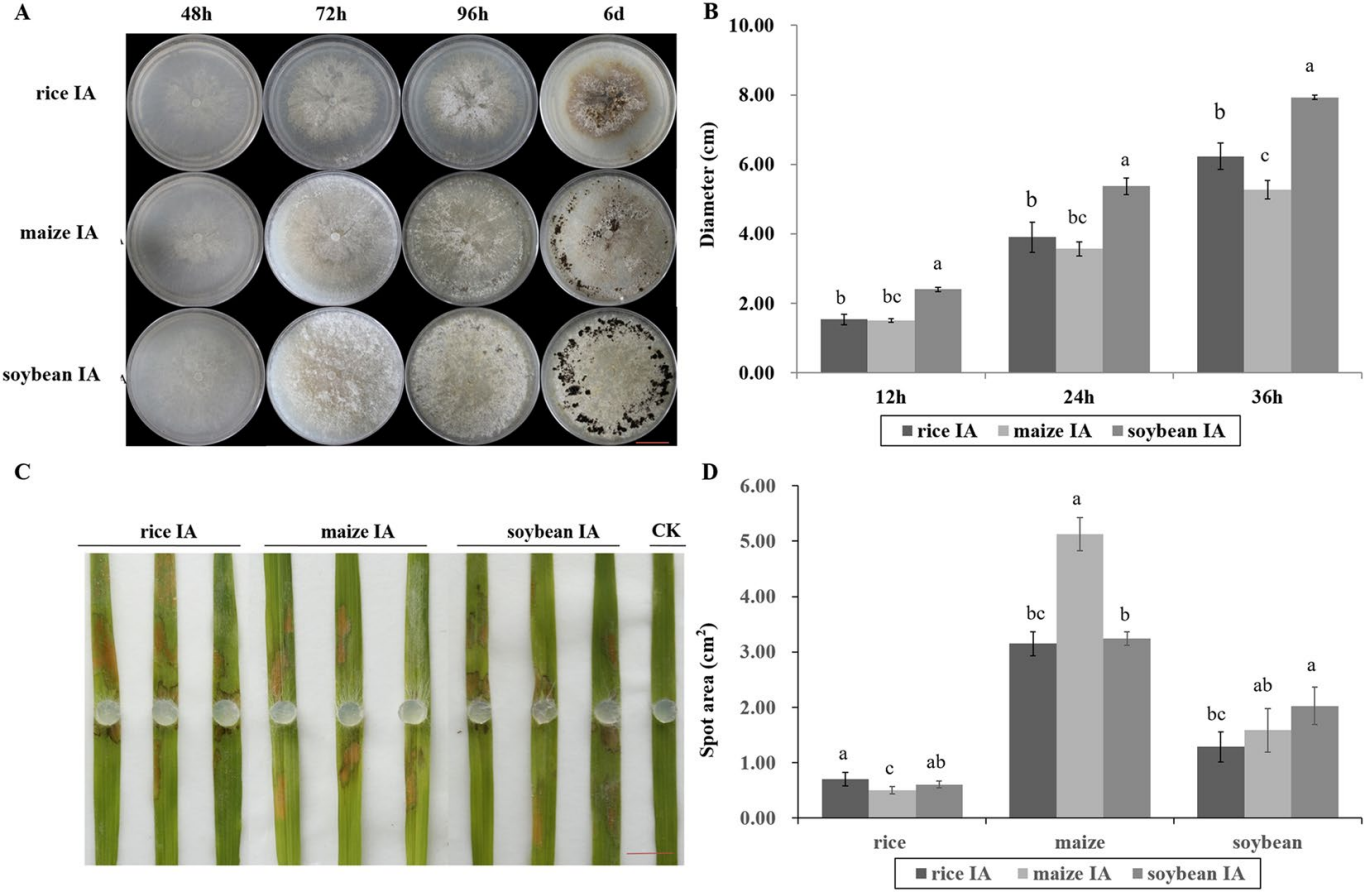
Biological characteristics of strains from different hosts. (**A**) Colony morphology of rice IA, maize IA and soybean IA after culture on PDA for 48, 72 and 96 h and 6 days. Scale bars, 2 cm. (**B**) Diameter of mycelia of rice IA, maize IA and soybean IA cultured on PDA at 12, 24 and 36 h post-inoculation (hpi). Error bars, ±standard error (SE) of means of raw data. The least significant difference (LSD) method was used; different lower case letters (**a**–**c**) indicate significant differences among three strains at P < 0.05. (**C**) Pathogenicity of rice IA, maize IA and soybean IA. Four-week-old rice cultivar 93–11 leaves were inoculated onto a block of PDA containing fresh medium (diameter 0.5 cm); controls were inoculated with fresh PDA. Typical leaves were photographed at 48 hpi. Scale bars, 1 cm. (**D**) Histogram depicting spot area caused by rice IA, maize IA and soybean IA under infection of rice, maize and soybean leaves at 48 hpi. Error bars, ± SE of means of raw data. The least significant difference (LSD) method was used; different lower case letters (**a**–**c**) indicate significant differences among three strains at P < 0.05.

For colony morphology on potato-dextrose agar (PDA) (Fig. 1A), the strains exhibited differences in sclerotium formation. Soybean IA formed the most aerial hyphae, followed by maize IA and rice IA. The sclerotia of *R. solani* are the primary source of sheath blight infection, and the number of sclerotia is a determinant of disease severity. The sclerotia formed by soybean IA were relatively large and distributed at the edge of the plate. Sclerotia formed by maize IA were not connected and distributed at the edge of the plate, with few located near the inoculation point. In contrast, rice IA formed a sclerotium near the inoculation point. The mycelium diameters of rice IA and maize IA, were not significantly different at the early stage (from 0 to 24 h after inoculation), but soybean IA showed significantly faster growth than the other two strains, from 12 to 36 h (Fig. 1B). To explore the differences in pathogenicity among the three *R*. *solani* AG1 IA strains isolated from rice, maize, and soybean, a detached-leaf inoculation method was used, that allows for convenient inoculation, spot amplification, and rapid disease development^38^. Differences in the area of spots were observed (Fig. 1C). Spots from rice leaves showed that the disease caused by rice IA was significantly more severe than that caused by maize IA but showed no significant difference with soybean IA, and there was no significant difference between maize IA and soybean IA at 48 h post-inoculation (hpi) (Fig. 1D). Furthermore, the same experiment was performed using maize and soybean as host plants at 48 h post-inoculation (hpi). Spot areas from maize leaves showed that the disease caused by maize IA was significantly more severe than that caused by rice IA and soybean IA and spots from soybean leaves also indicated that the disease caused by soybean IA was significantly more severe than rice IA. However, no significant difference was found between soybean IA and maize IA (Fig. 1D, Supplementary Fig. S2). These results show that *R. solani* AG1IA strains isolated from different host plants exhibit different levels of pathogenicity and show greater pathogenicity to their original hosts.

### Transcriptome sequencing and analysis

To gain insight into the pathogenesis of *R. solani* AG1IA in different hosts, transcriptomes of the abovementioned strains with the same infection stage (infection structures formed) in different hosts were sequenced using Illumina NGS technology; strains without host infection were used as controls. In total, 104.97 million paired-end reads with Q30 > 85%, which indicated raw data of high quality, were obtained (Supplementary Table S1). Of the short clean reads, 68.94–79.31% were mapped onto the *R. solani* AG1IA reference genome^28^, and mapped reads were further classified and annotated using TopHat^39^ (Supplementary Table S2). Overall, 9,077, 9,106, 8,850, 8,878, 8,923, 8,997, 8,943 and 8,913 genes were identified in R-R (rice IA during rice infection), R0 (rice IA grown on PDA), M-M (maize IA during maize infection), M0 (maize IA grown on PDA), M-R (maize IA during rice infection), S-S (soybean IA during soybean infection), S0 (soybean IA grown on PDA), and S-R (soybean IA during rice infection) (FPKM value > 0.5), respectively. In addition, 1,840 novel genes named Rice_newGene_999 were discovered and assembled using Cufflinks^40^ after filtering out short encoded peptides (<50 amino acid residues) and genes containing only a single exon. Among the novel genes, 11% were successfully annotated in KEGG (Kyoto Encyclopedia of Genes and Genomes), 26.9% in gene ontology (GO), 27% in COG, 41.5% in SWISS-PROT, and 85% in NR (Supplementary Table. S3). Twelve genes were selected randomly for quantitative polymerase chain reaction (qPCR) analysis, and most of the qPCR results were consistent with those of RNA-Seq data (Supplementary Fig. S3). Thus, the RNA-Seq data are reliable.

### Differentially expressed genes during host infection

**Figure 2:**
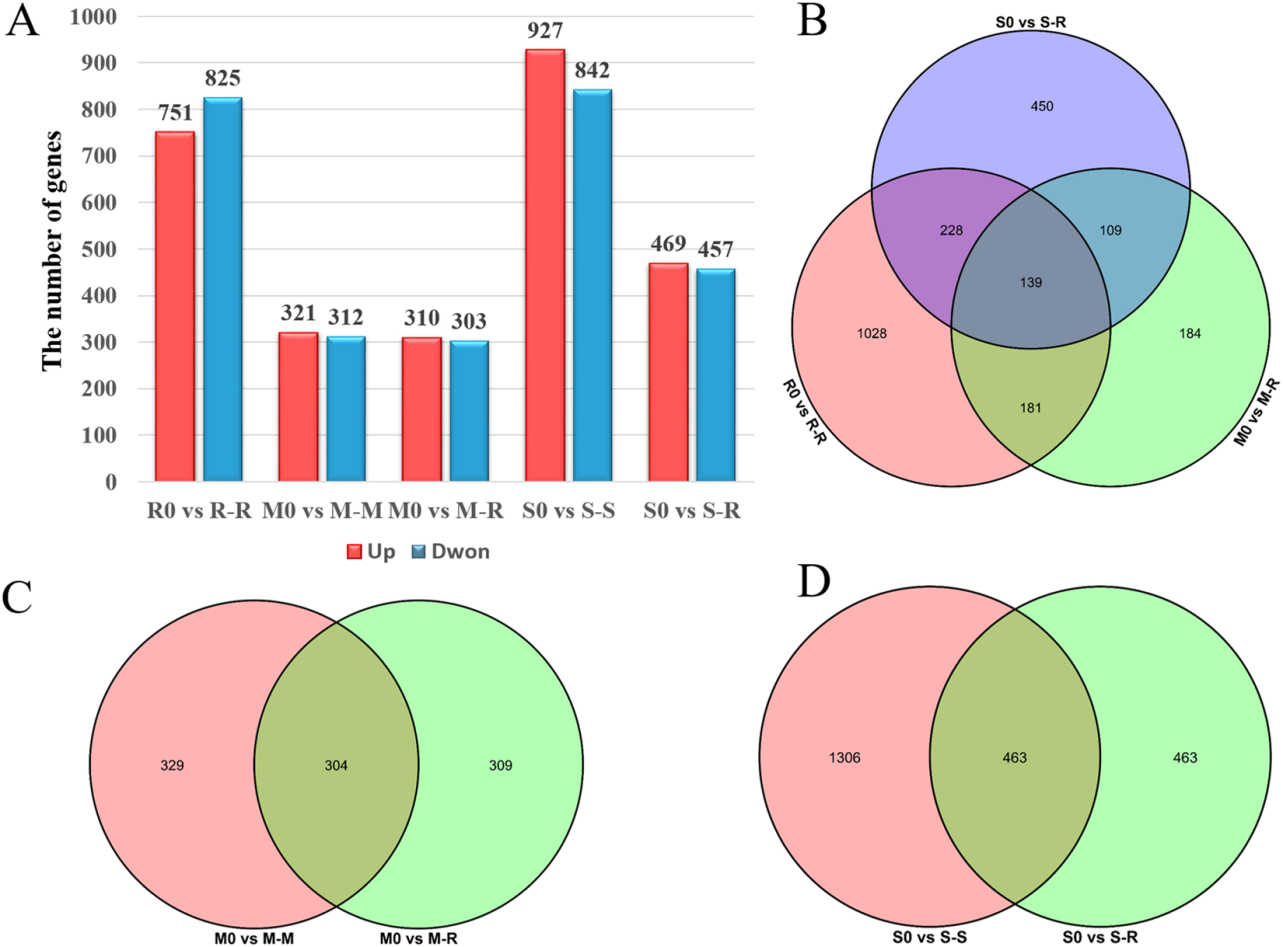
Overview of serial analysis of DEGs identified by pairwise comparison of eight transcriptomes. In total, 2,319 DEGs were found during different host infections with a |log2FC| ≥ 1and FDR < 0.05. (**A**) Numbers of DEGs identified in R0 vs. R-R, M0 vs. M-M, M0 vs. M-R, S0 vs. S-S and S0 vs. S-R. (**B**) Venn diagrams of DEGs in three strains during infection of rice. (**C**) Venn diagrams of DEGs in maize IA during infection of rice and maize. (**D**) Venn diagrams of DEGs in soybean IA during infection of rice and soybean.

A detailed comparative analysis of differentially expressed genes (DEGs) was performed to investigate the mechanisms underlying the pathogenesis of *R. solani* AG1IA during the infection of various hosts. A total of 2,319 DEGs were detected and comparison of the strains during rice infection resulted in the identification of 1,576, 613, and 926 DEGs in R0 vs. R-R, M0 vs. M-R, and S0 vs. S-R (Fig. 2A), respectively. Therefore, during infection of rice, more genes were involved in the regulation of rice IA than in maize IA and soybean IA. Surprisingly, few DEGs (139, 5.99%) were common to the three strains; 1,028, 184, and 450 genes were specifically regulated in R-R, M-R, and S-R, respectively, which accounts for 71.67% of the total (Fig. 2B). During infection of the same host (rice), the gene expression profiles of *R. solani* strains varied markedly, suggesting the co-evolution of strains with their original hosts. Next, DEGs in strains of different ecotypes during infection of the original host and non-original host (rice) were analysed. During infection of rice and maize by maize IA, a total of 942 DEGs were detected, 304 were commonly expressed, and 329 and 309 DEGs were specifically expressed in M-M and M-R, respectively (Fig. 2C). In contrast, 2,232 DEGs were detected in S-S and S-R; specifically, 1,306 and 463 genes were regulated in S-S and S-R, respectively and 463 DEGs were commonly expressed (Fig. 2D). Interestingly, the total number of DEGs in S-S and S-R was markedly higher than in M-R and M-M, suggesting that more DEGs participated in soybean IA infection. More DEGs were found upon infection of the original host than the non-original host in maize IA and soybean IA. These results suggest the gene expression pattern of *R. solani* AG1IA differed according to the host plant and possesses considerable host-adaptation ability, which could likely explain its wide host range.

### Gene ontology enrichment analysis of DEGs

**Figure 3:**
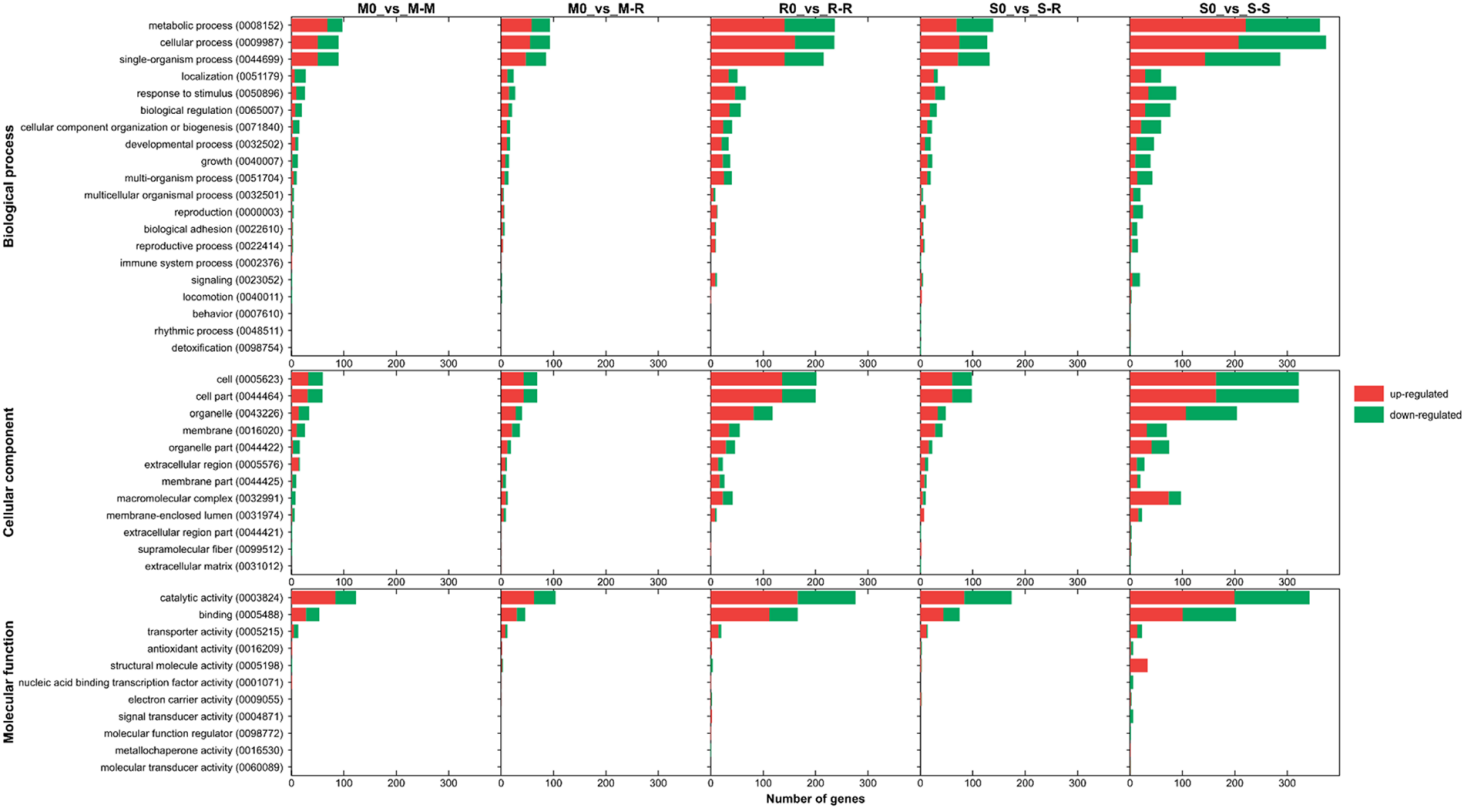
Numbers of GO categories with increased and decreased mRNA levels. Statistically significant data were grouped into broad GO categories (by TAIR). Bars represent number of up-regulated (red) and down-regulated (green) probe sets.

Gene ontology (GO) enrichment analysis of cDNA libraries was performed to classify the function of DEGs during host infection and to gain insight into their involvement in biological, molecular, and cellular processes. To obtain a global view of differences in the transcriptome, the number of GO categories with increased and decreased mRNA levels was compared (Fig. 3). Among them, cellular processes, metabolic processes, single-organism processes, cell part, cell and catalytic activity accounted for the majority of DEGs in all comparisons. In addition, metabolic processes and catalytic activity were both pathogenicity-associated. Overall, 185 genes related to catalytic activity were up-regulated in R-R, almost three-fold higher than M-R, for which only 63 genes were up-regulated in this term. In addition, 199 genes of S-S were up-regulated in catalytic activity, almost two-fold the amount of S-R. Surprisingly, 220 genes for S-S were up-regulated in metabolic processes, while only 74 genes were up-regulated in S-R, similar to the pattern for other processes (e.g., cellular processes, single-organism processes, cell part, cell). Differential expression of up-regulated and down-regulated genes in pathogenicity-associated categories suggests more complex mechanisms of gene regulation during infection of the original host than the non-original host in rice IA and soybean IA.

**Figure 4:**
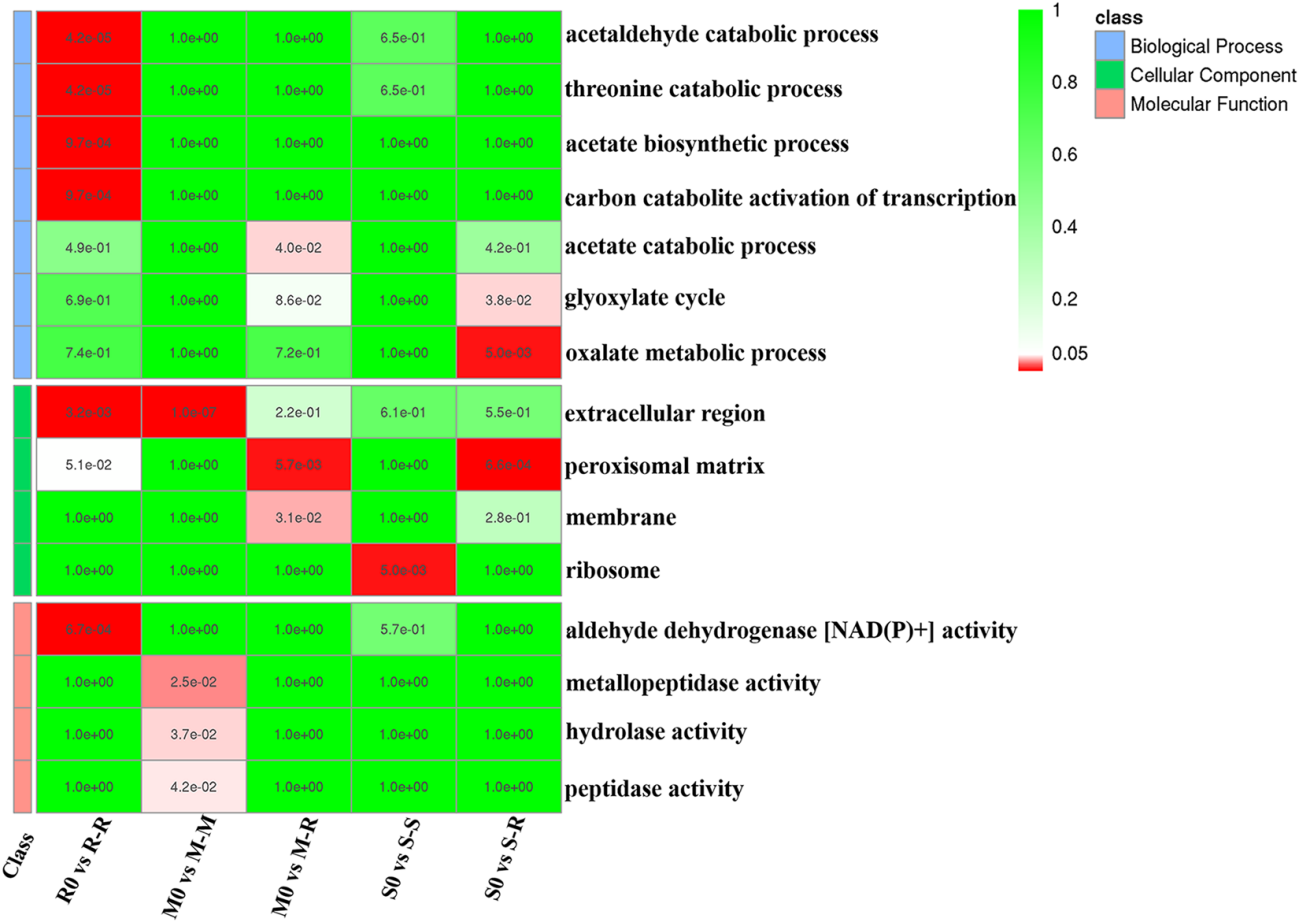
GO term enrichment analysis of strains during infection of different hosts. GO terms were enriched with a Hypergeometric test, and q-values were adjusted with FDR < 0.05. Scale represents p-value after homogenization.

Analysis of R-R, M-R, and S-R during rice infection showed that the GO terms ‘acetaldehyde catabolic process,’ ‘extracellular region,’ and ‘aldehyde dehydrogenase activity’ were significantly (q < 0.05) enriched in R-R (Fig. 4). However, ‘peroxisomal matrix,’ which is closely related to the pathogenicity of filamentous fungi, was significantly enriched in M-R and S-R, and ‘oxalate metabolic process’ was only highly significantly enriched in S-R. The oxalate metabolic process is associated with pathogenicity because several pathogens (e.g., *M. oryzae* and *F. graminearum*) secrete oxalate to acidify host tissues and sequester calcium from host cell walls^41, 42^. The GO term ‘extracellular region’ (associated with secretion) was highly significantly enriched in M-M compared to M-R, whereas ‘peroxisomal matrix’ was highly significantly enriched in M-R compared to M-M (Fig. 4). The GO terms of soybean IA during infection of soybean and rice were compared. The results showed that ‘ribosome’ (associated with thermotolerance and virulence in pathogenic fungi^43^) was highly significantly enriched in S-S, and ‘oxalate metabolic process’ and ‘peroxisomal matrix’ were highly significantly enriched in S-R (Fig. 4). Therefore, ‘peroxisomal matrix’ was the most abundant term in all strains during rice infection. In contrast, ‘extracellular region’ was the most abundant term used during maize infection. These results show that, although all three strains belong to the AGIIA subgroup, the GO terms (particularly those associated with pathogenicity) enriched in DEGs differ considerably according to host plant.

### Alternative splicing

A comprehensive analysis of alternative splicing events in eight *R. solani* AG1IA transcriptomes was performed using high-throughput RNA-seq. A total of 11,341(4,462), 11,030 (4,471), 10,871 (4,465), 10,986 (4,355), 11,126 (4,468), 10,619 (4,381), 11,020 (4,463) and 10,794 (4,402) alternative splicing events (genes) of six AS types (FPKM > 0.5) were detected in R-R, R0, M-M, M0, M-R, S-S, S0, and S-R, respectively (Supplementary Table S4). Moreover, an average of 49.5% of genes in *R. solani* AG1IA underwent alternative splicing in this study. To the best of our knowledge, this is the first study to report a large number of alternative splicing events and a high proportion of AS genes in *Rhizoctonia*, both of which are markedly higher than the equivalent values for *R. solani* AG1IB^32^. The following six classes of alternative splicing were predicted among the eight transcriptomes: exon skipping, intron retention, alternative 5′ splice site, alternative 3′ splice site, alternative first exon and alternative last exon (Supplementary Fig. S4). Intron retention was the most abundant type of alternative splicing. For which *AG1IA_03552* was predicted as a candidate effector in *R. solani* AG1IA^28^ was identified alternative spliced in R-R and the splice variant were obtained for further research on the pathogenic mechanisms affected by alternative splicing (Supplementary Fig. S5).

Alternative splicing likely regulates the virulence and gene expression of pathogenic fungi^44–46^. In previous studies, secreted proteins, CAZymes, cysteine-rich proteins, ABC transporters, and G protein-coupled receptors (GPCRs) have been identified as pathogenesis-associated factors in *R. solani* AG1IA which involved in pathogenicity^28^. Therefore, we performed statistical analysis of the number of alternative splicing events in these five pathogenesis-associated factors which showed an average proportion of 16.41% have been predicted to be alternative spliced (Supplementary Table S5). Intron retention was the most abundant type of alternative splicing of genes encoding pathogenesis-associated factors. The AS events in above mentioned factors might suggest the development of new pathogenic mechanisms during the co-evolution of strains with their hosts.

**Figure 5:**
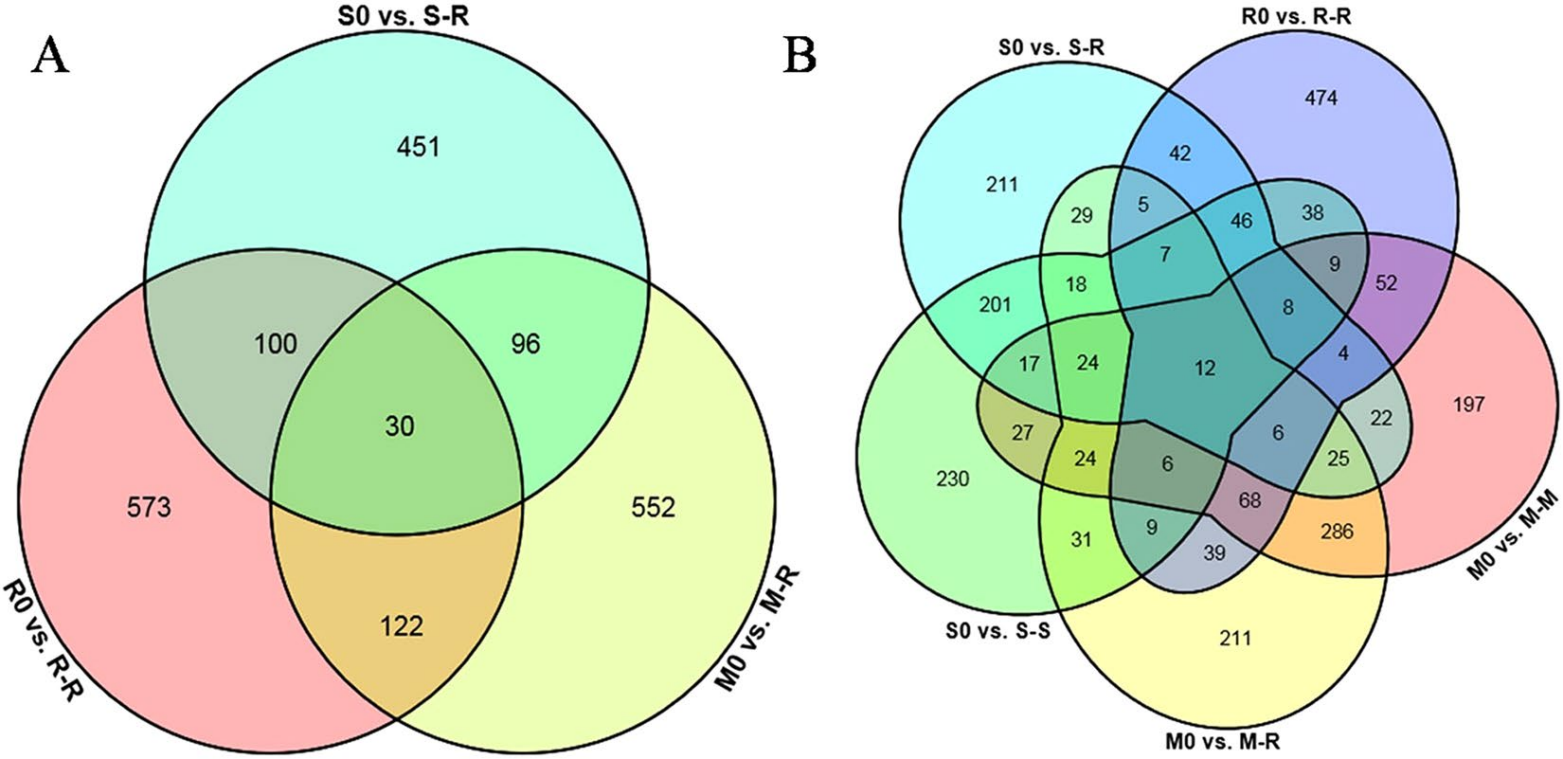
Genes underwent alternative splicing only during host infection compared to vegetative mycelia. (**A**) Venn diagrams of genes underwent alternative splicing only during infection of rice. (**B**) Venn diagrams of genes underwent alternative splicing during infection of rice, maize and soybean.

The genes that underwent alternative splicing only during host infection compared with non-host (vegetative mycelia) were subjected to further analysis. Surprisingly, during rice infection, 573, 552, and 451 genes underwent alternative splicing in R0 vs. R-R, M0 vs. M-R, and S0 vs. S-R, specifically and respectively, whereas 30 genes underwent alternative splicing in all three comparisons (Fig. 5A). Among the 30 common AS genes, three genes (*AG1IA_06529*, *AG1IA_05348*, and *AG1IA_02459*) were annotated in the PHI database. Surprisingly, twelve genes underwent alternative splicing during infection of all three hosts, eight of which were up-regulated (Fig. 5B). Among these eight genes, except for one hypothetical protein (*AG1IA_01513*), seven were predicted to be related to pathogenicity, including a cytochrome P450-domain-containing protein (*AG1IA_00285*) and a glycoside hydrolase family 16 protein (*AG1IA_09291*) (Supplementary Table S6). Therefore, genes that undergo alternative splicing may play critical roles in the regulation of gene expression, the infection process and environmental adaptation by *R. solani* AG1IA. Further research on these candidate genes should seek to determine how their regulation via alternative splicing regulates the virulence of *R. solani* AG1IA.

### Secreted proteins and effectors

**Figure 6:**
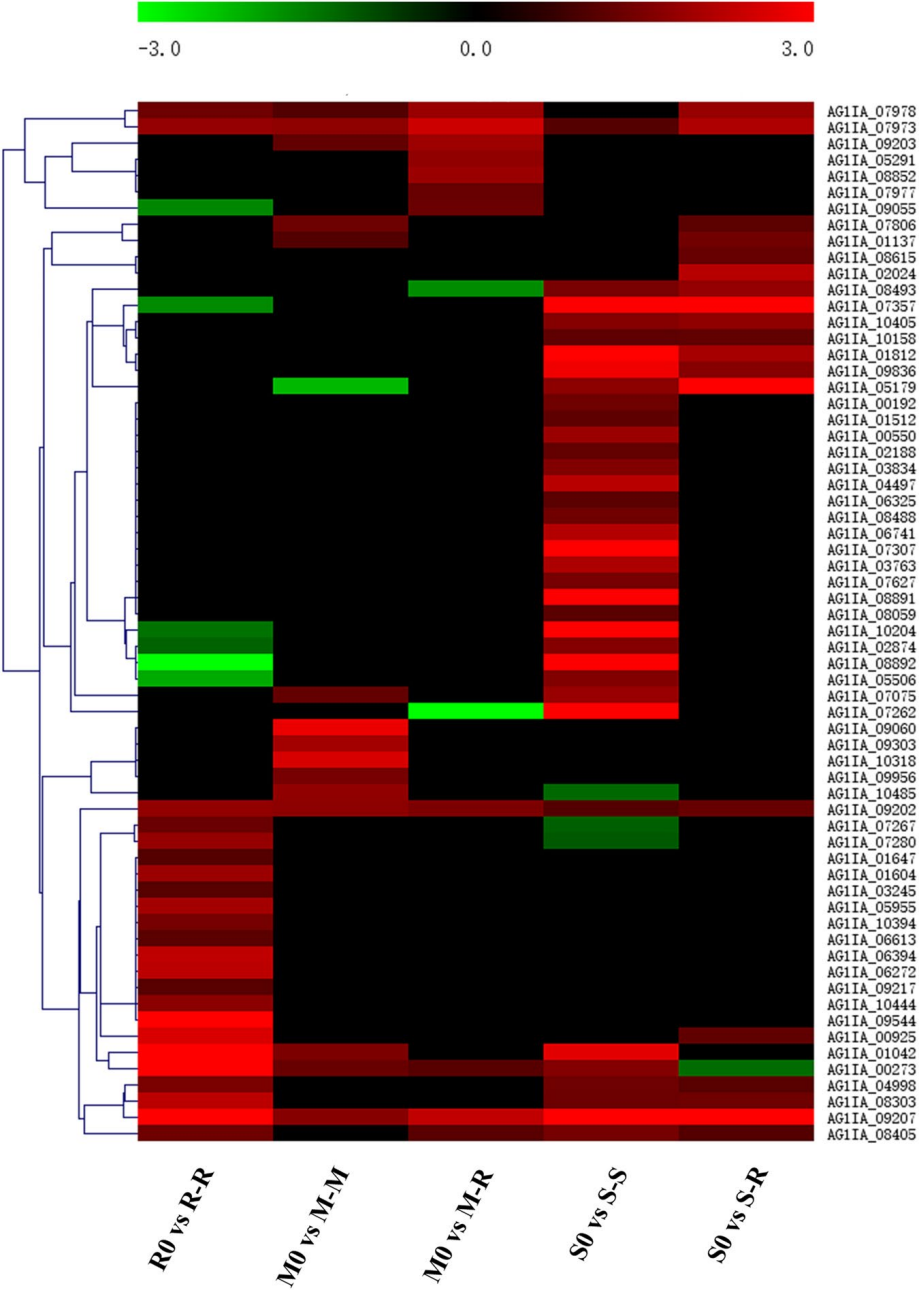
Heat map of up-regulated candidate secreted effectors during infection of various hosts. Sixty-four up-regulated candidate secreted effectors of *R. solani* AG1IA were analysed by prediction algorithms; a hierarchical clustering statistic method based on Pearson’s correlation (correlation ≥ 98%) and average linkage was used. Red indicates a significant increase in gene expression, black indicates no change in expression and green indicates a significant decrease in gene expression (|log2FC| ≥ 1). Colour scale represents log_2_ fold change values from -3 to 3.

We also focused on known pathogenicity-related genes in *R. solani* AG1IA including those encoding secreted proteins, or candidate effectors^28^. Overall, 347 secreted proteins were differentially expressed during the infection of various hosts (Supplementary Fig. S6). Among them, six genes (*AG1IA_06531*, *AG1IA_09202*, *AG1IA_07681*, *AG1IA_07973*, *AG1IA_09207* and *AG1IA_09720*) with two genes (*AG1IA_09369* and *AG1IA_10043*) were up-regulated and down-regulated under all infection conditions, respectively. The six genes up-regulated under host infection conditions of different hosts are likely to play an important role in pathogenic mechanisms in the PHI and could be used for further research. Among them, the up-regulation of *AG1IA_09202* under different infection conditions was confirmed through qRT-PCR (Supplementary Fig. S3). Effectors are known to contribute to disease establishment in the host, disturbing host plant physiology, and most are secreted proteins. In this study, effector P (http://effectorp.csiro.au/) was used to predict candidate secreted effectors among secreted proteins^47^. A total of 104 candidate effectors were differentially expressed (Supplementary Table S7), and 64 up-regulated candidate secreted effectors of *R. solani* AG1IA were further analysed (Fig. 6). Among them, most genes showed different expression patterns under different host infections; in contrast, three genes (*AG1IA_07973*, *AG1IA_09202* and *AG1IA_04998*) were significantly up-regulated, but only *AG1IA_10043* was significantly down-regulated under all infection conditions. Notably, 23, 15, 11, 35 and 19 genes were up-regulated in R-R, M-M, M-R, S-S and S-R, respectively. According to our data, the number of up-regulated candidate effectors was higher in the original hosts than non-original hosts, as particularly evident in rice IA and soybean IA. Among these up-regulated candidate effectors, 13, 5, 4, 18 and 4 genes were differentially and specifically up-regulated in R-R, M-M, M-R, S-S and S-R, respectively. Furthermore, we analysed the function of these 104 candidate effectors; only 27 genes were found to have predictable functions, while 77 candidate effectors had no homology to known effectors in the NCBI database and were annotated as hypothetical proteins in this study. Additionally, 17 genes of the abovementioned 27 genes were up-regulated during host infection (Supplementary Table S8).

### Expression of key hydrolases

**Figure 7:**
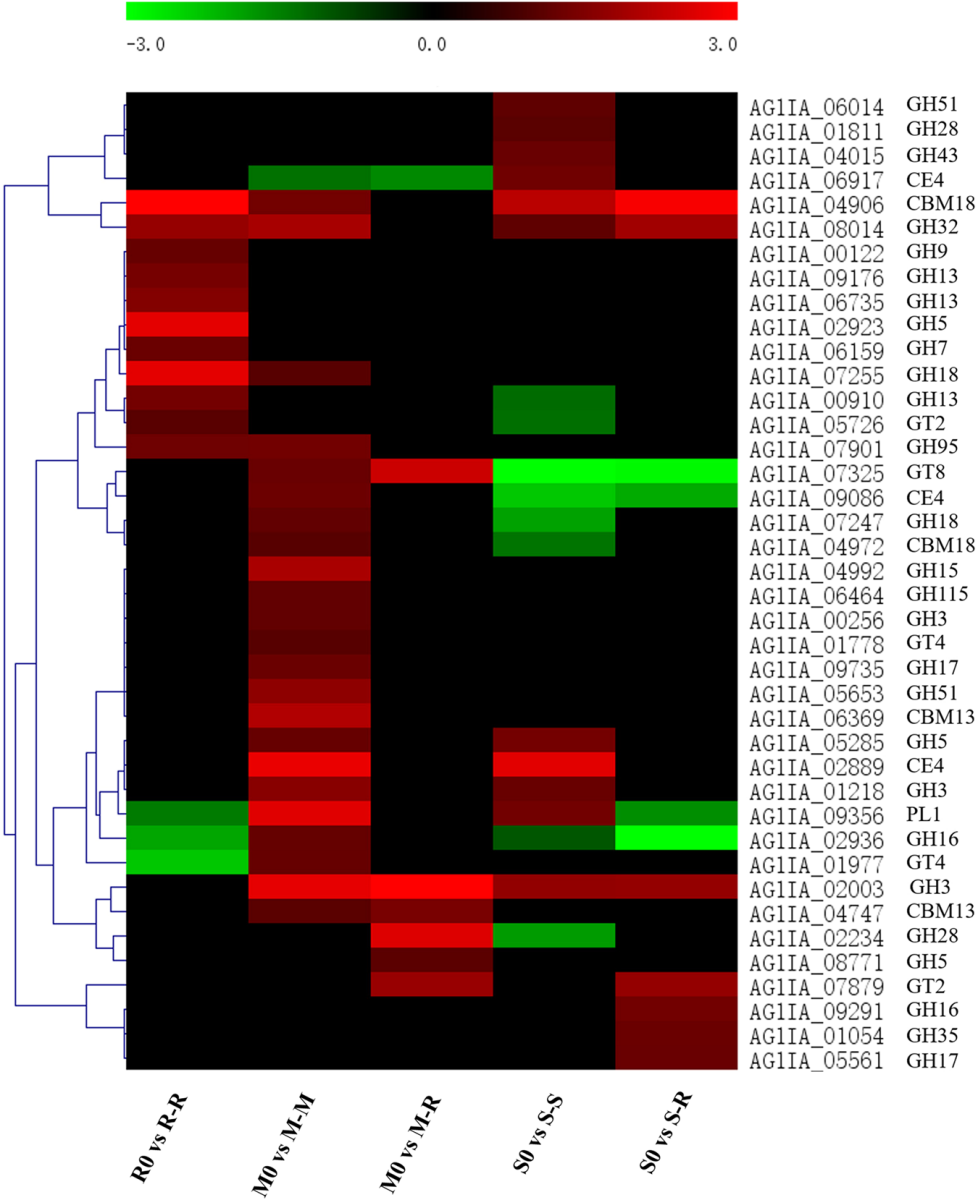
CAZyme family genes expressed in rice IA, maize IA and soybean IA during infection of various hosts. In total, 40 up-regulated genes were clustered; of these, 29 genes showed a significant host-specific up-regulated expression pattern during the infection of different hosts. A hierarchical clustering statistic method based on Pearson’s correlation (correlation ≥ 98%) and average linkage was used. Red indicates a significant increase in gene expression, black indicates no change in expression and green indicates a significant decrease in gene expression (|log2FC| ≥ 1). Colour scale represents log_2_ fold change values from −3 to 3.

CAZymes, which include glycosyl transferases (GTs), glycoside hydrolases (GHs), polysaccharide lyases (PLs), carbohydrate esterases (CEs) and non-catalytic carbohydrate-binding modules (CBMs), have been a focus of many research studies because of their importance in the penetration and successful infection by fungal pathogens^28^. In this study, 40 putative CAZymes of DEGs, which were up-regulated during the infection of various hosts, were further analysed and are likely to be involved in plant infection or survival (Fig. 7). In total, 29 genes (7 in R-R, 12 in M-M, 3 in M-R, 4 in S-S and 3 in S-R) were specifically up-regulated, and 6 genes (1 in R-R and 5 in S-S) were specifically down-regulated. During the infection of rice, all up-regulated DEGs coding for CAZymes in R-R, M-R and S-R belonged to the GH, CBM and GT families, but no DEGs belonged to the CE families. Interestingly, eleven putative CAZymes in R-R were up-regulated which were more than in M-R and S-R. Twenty-three putative CAZymes were up-regulated during M-M infection, the highest for any conditions and nearly four-fold higher than M-R. Similarly, the number of putative CAZymes up-regulated in maize IA during maize infection was nearly two-fold higher than during rice infection. However, the number of up-regulated CAZymes in S-S was also nearly 1.5-fold that of S-R. From the data above, more up-regulated hydrolase genes participate in the infection of their original host compared to the non-original host. Interestingly, some CAZymes up-regulated in R-R and M-M were significantly down-regulated in S-S, which might indicate different gene regulation patterns for CAZymes in rice IA, maize IA and soybean IA. To better understand the pathogenesis of CAZymes during infection of different hosts, up-regulated genes encoding putative enzymes involved in the degradation of plant cell wall components (mainly GHs and PLs) were further classified (Supplementary Table S9). We conclude that CAZymes that mediate cellulose, hemicellulose and starch could be more necessary during rice infection. Therefore, CAZymes that mediate the degradation of celluloses, hemicellulose and chitin could be likely more required during maize infection, and those that mediate pectin degradation could be more important during soybean infection.

### Secondary metabolites

**Figure 8:**
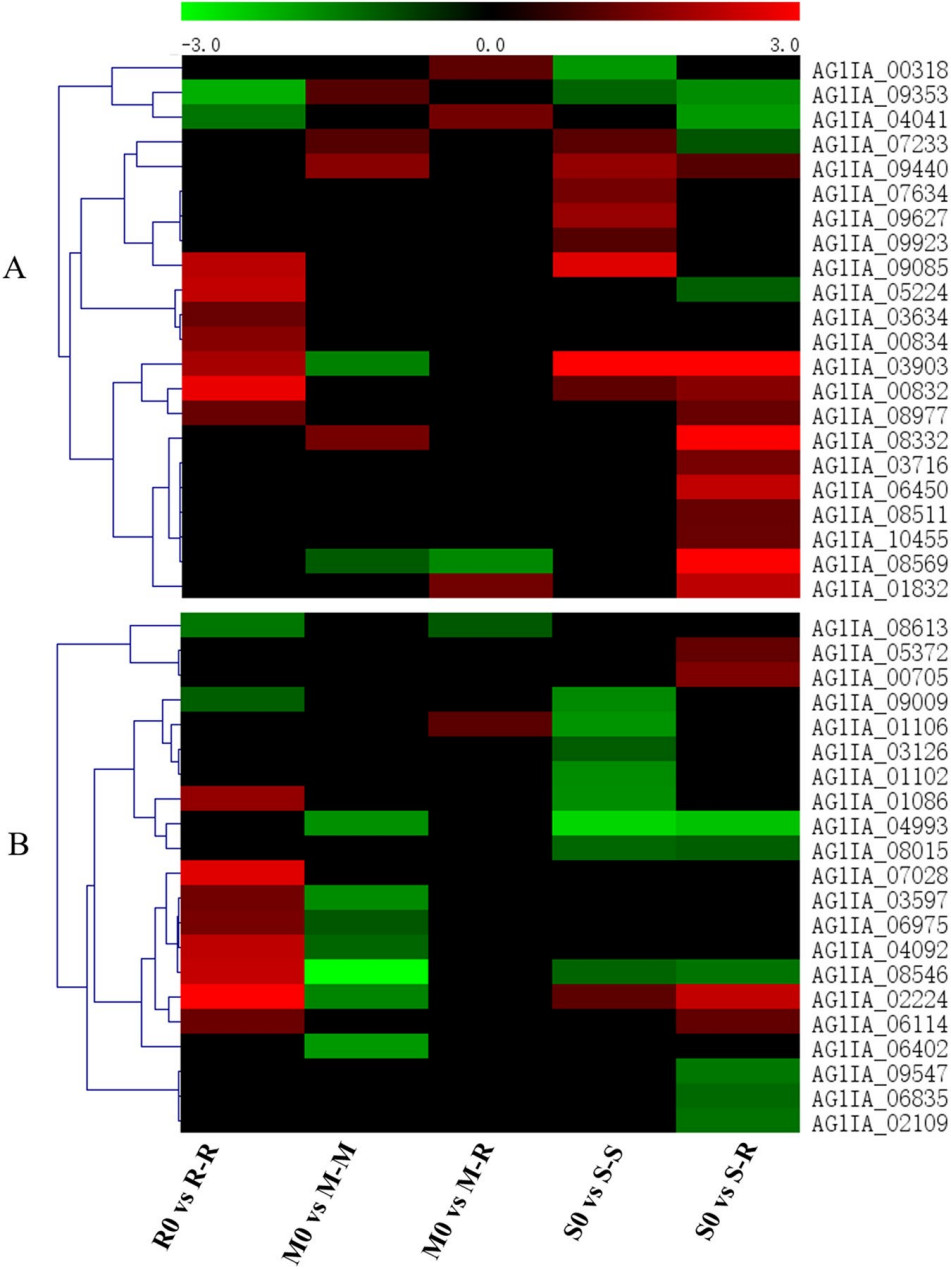
Differential expression of secondary metabolite-associated genes in *R. solani* AG1IA during infection of various hosts. (**A**) Expression profiles of up-regulated cytochrome P450 during infection of various hosts. (**B**) Expression profiles of differentially expressed candidate ABC transporters during infection of various hosts. A hierarchical clustering statistic method based on Pearson’s correlation (correlation ≥ 98%) and average linkage was used. Red indicates a significant increase in gene expression, black indicates no change in expression and green indicates a significant decrease in gene expression (|log2FC| ≥ 1). Colour scale represents log_2_ fold change values from −3 to 3.

Fungal pathogens generally produce an array of secondary metabolites, some of which are involved in pathogenesis, and their production is frequently correlated with a specific stage of morphological differentiation^48^. The cytochrome P450 (CYP) monooxygenases of fungi are involved in many essential cellular processes and in primary and secondary metabolic pathways during host infection^49^. Sixty-eight putative CYPs were identified in the *R. solani* AG1IA genome, and are likely to play a role in the metabolism and biotransformation of compounds toxic to the host^28^. Among them, twenty-two CYPs were differentially up-regulated during the infection of different hosts (Fig. 8A). As seen from the heat map, rice IA and soybean IA required a larger number of up-regulated CYPs during infection, whereas maize IA required relatively few. Therefore, CYP genes differentially expressed during infection likely play different roles in the metabolism and biotransformation of compounds toxic to the host.

Transporters export toxic molecules to the surrounding environment^50^. In toxin-producing fungi, transporters mediate the efflux of endogenously produced molecules. Among them, ABC transporters in plant pathogenic fungi are involved in the transport of plant-defense compounds and fungal pathogenicity factors. *R. solani* AG1IA is predicted to harbour 48 ABC transporters^28^. In this study, 21 ABC transporters were differentially expressed during the infection of different hosts (Fig. 8B). More DEGs encoding ABC transporters were up-regulated in R-R compared to M-R and S-R. Surprisingly, no ABC transporters were significantly up-regulated in M-M, but five were specifically down-regulated, similar to S-S. Therefore, our data suggest that ABC transporters could be more important during infection of rice than of maize or soybean.

### Proteins related to the MAPK pathway

**Figure 9:**
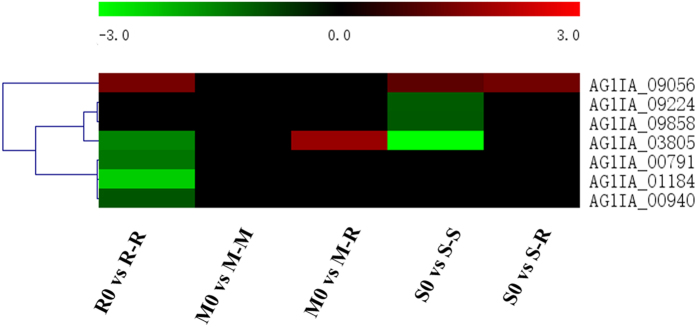
Differential expression of G-protein-coupled receptors (GPCRs) in *R. solani* AG1IA during infection of various hosts. Seven DEGs of G-protein-coupled receptors (GPCRs) were analysed by prediction algorithms using the hierarchical clustering statistic method based on Pearson’s correlation (correlation ≥ 98%) and average linkage. Red indicates a significant increase in gene expression, black indicates no change in expression and green indicates a significant decrease in gene expression (|log2FC| ≥ 1). Colour scale represents log_2_ fold change values from −3 to 3.

G-protein-coupled receptors (GPCRs) are involved in fungi-plant interactions and in the regulation of morphogenesis, mating, infection, and virulence in plant-pathogenic fungi^51, 52^. Thirteen GPCR-like genes have been identified in *R. solani* AG1IA^28^. In this study, seven predicted GPCR-like DEGs were found during the infection of different hosts (Fig. 9). Interestingly, *AG1IA_09056*, which was predicted to contain a NCD3G nine-cysteine domain, was found to be up-regulated in R-R, S-S and S-R. *AG1IA_03805*, which is a predicted homologue of *Homo sapiens* mPR-like GPCRs, was up-regulated in M-R but down-regulated in R-R and S-S. *AG1IA_00940*, *AG1IA_01184* and *AG1IA_00791* were significantly and specifically down-regulated in R-R. *AG1IA_00940* was predicted as an RGS (Regulator of G-protein Signaling) domain-containing protein which play pivotal roles in upstream regulation of fundamental biological processes in fungi, including vegetative growth, sporulation, mycotoxin/pigment production, pathogenicity, and mating^53^. While *AG1IA_01184* was predicted to be a stm1-related protein which have been verified to participate in apoptosis-like cell death in yeast^54^. These differentially expressed GPCR genes could also reflect the adaptation of *R. solani* AG1IA to different host plants.

## Discussion

Host selection affects the population genetic structure of *R. solani* AG1IA, and the pathogenicity of *R. solani* strains of different groups may differ among host plants^36, 55, 56^. Rice IA, maize IA, and soybean IA were isolated from Xichang (altitude of 1500 m), Chengdu (altitude of 500 m) and Yaan (altitude of 1000 m), respectively. Hyphal fusion is only permitted between genetically similar heterokaryons sharing the same allele specificities at all *het* loci. When two interacting heterokaryons are genetically dissimilar, with different allelic specificities at some or all *het* loci, interaction hyphae cell death prevents dissimilar individuals from anastomosing^57–59^. Different ecotypes of rice IA, maize IA, and soybean IA could not anastomose with each other, indicating that they are genetically different. Cytological and morphological differences further suggested that the long-term co-evolution of strains with their host plants led to the generation of genetic differences. Rice IA, maize IA, and soybean IA can infect rice, but were more aggressive towards their original hosts, consistent with host specialization. Although *R. solani* AG1IA is a soil-borne fungus that can infect a broad range of hosts, each strain has adapted to its host in terms of morphology, physiology, and biochemistry. Moreover, rice is mainly cultivated in regions of high temperature and high humidity, whereas maize and soybean are mainly cultivated in arid regions. The abovementioned strains exist in specific niches in their various hosts due to ecological adaptation and long-term evolutionary selection.

Transcriptomic analyses of the strains during infection of different hosts were performed using Illumina next-generation sequencing. To the best of our knowledge, this is the first study to use comparative transcriptome profiling analysis to explore the pathogenic mechanisms of *R. solani* AG1IA during infection of different hosts. The currently available sequences enhance our understanding of the transcription profiles of *R. solani* AG1IA ecotypes during the infection of different hosts. In fact, the differences between our 25 sequenced strains from rice ecological cultivation areas were very small (unpublished). However, differences in the proportions of reads mapped in rice IA, maize IA and soybean IA indicated that their genomic sequences differ markedly.

To evaluate the pathogenic mechanisms of different strains of *R. solani* AG1IA during the infection of different hosts, the transcriptomes of the three strains were compared during the infection their original and non-original hosts. DEGs in R-R were significantly involved in acetaldehyde catabolism, the extracellular region and aldehyde dehydrogenase [NAD(P)^+^] activity, unlike the DEGs in M-R and S-R. In particular, aldehyde dehydrogenase is associated with pathogenesis and plays important roles in normal physiological processes and in response to stressors^60^. Pathogenic bacteria overcome the oxidative stress generated by host immune effectors by expressing a variety of aldehyde dehydrogenases^61^. What’s more, DEGs in S-R were significantly involved in oxalate metabolism and the peroxisomal matrix, which markedly differs from the roles of DEGs in S-S. The peroxisome is closely related to the pathogenicity of plant-pathogenic fungi, particularly filamentous fungi, by influencing fat metabolism and melanin synthesis^62, 63^. Peroxisome metabolism affects invasion by many plant pathogens^64^. Interestingly, DEGs involved in the peroxisomal matrix were only significantly enriched during the infection of rice (R0 vs. R-R, M0 vs. M-R and S0 vs. S-R). Therefore, we hypothesized that the pathogenic mechanism of *R. solani* AG1IA is closely associated with the host plant.

Splicing in general, and alternative splicing in particular, is important for the regulation of gene expression and disease pathogenesis^65^. RNA-seq has revealed that a high proportion of multiexonic genes (>42.4% in rice^66^, 61% in *Arabidopsis* ^67^ and 95% in humans^68^) undergo alternative splicing. In contrast, a previous study revealed that an average of 6.4% of fungal genes are affected by this process^46^ while the proportion of alternatively spliced (AS) genes in *Trichoderma longibrachiatum* ^69^ is markedly higher (48.9%) than in *M. grisea* (1.6%)^70^, *Ustilago maydis* (3.6%)^71^ and *A. oryzae* (8.55%)^72^, suggesting that alternative splicing events in fungi are far more frequent than has been reported and could play an important role in fungal transcriptome diversity. Several proteins arising from specific AS transcripts have been reported, and genes with alternative splicing events were used to develop a transposon tagging system that was verified in a fungal pathogen^73–75^. Alternative splicing events are developmentally regulated and associated with responses to various environmental conditions^76, 77^, the unexpectedly high proportion of AS genes especially pathogenesis-associated genes suggests that alternative splicing could be an important mechanism in the gene regulation of *R. solani* AG1IA. Further investigations are needed to confirm whether *R. solani* AG1IA produces a large number of AS forms to adapt to different host infections which might explain its wide host range. However, previous studies have confirmed the relationship between alternative splicing and fungal pathogenicity. For example, a virulence gene (UrRm75) in *U. maydis* and a ste12-like transcription factor that was essential for growth and pseudohyphal development in many fungi have also been reported to be alternatively spliced^78, 79^. The eight genes predicted to be up-regulated during all host infections will be utilized in further research on the role of alternative splicing in the pathogenesis or adaption of *R. solani* AG1IA during different host infections.

Non-toxic plant fungal proteins are predicted to be secreted proteins^80^, namely virulence factors that facilitate colonization during PHIs. These secreted proteins likely constitute the majority of effectors that elicit host responses and mediate the ability of fungi to perceive and respond to the environment^81, 82^. Secretion of effector proteins by plant pathogens such as rust fungi^83^ and *Magnaporthe oryzae* ^84^ into host tissues promotes infection by manipulating host processes. There are few reports on the effectors of *Rhizoctonia solani*, although 25 candidate effectors were predicted in *R. solani* AG1IA four years ago^28^. Surprisingly, nearly half of the candidate effectors (44 genes out of 104 genes) were found to be specifically up-regulated under different host infections and 17 have homology protein domain with known effectors. Indeed, effectors may have undergone rapid evolutionary changes, which may account for the varying virulence of the various AGs, ISGs, and ecotypes of *R. solani*, as well as their ability to infect a wide range of plants^28^. For example, *AG1IA_07075*, which was predicted as a CFEM domain-containing protein, was only up-regulated in M-M and S-S but not during rice infection. CFEMs are domains containing eight cysteines (e.g., Pth11 from *Magnaporthe grisea*) and are proposed to have important roles in fungal pathogenesis^85^. In contrast, *AG1IA_03245*, which was predicted to be a kinase domain-containing protein, was only up-regulated in R-R; *AG1IA_08303*, which was predicted as a peroxidase, family 2 domain-containing protein, was only up-regulated in rice IA and soybean IA. Thus, the differentially expressed candidate effectors are likely to be key factors in varying pathogenicity among host infections in *Rhizoctonia solani* AG1IA.

There is a clear relationship between CAZymes and the infection process in fungi^22, 86–88^. Many CAZymes are involved in the biosynthesis, modification, binding, and catabolism of carbohydrates. Among them, CAZymes of GHs and PLs are cell wall-degrading enzymes that play important roles in plant biomass decomposition by fungi and bacteria^89^. Pathogens secrete numerous cell wall-degrading enzymes that breach the plant cell wall and use them as sources of nutrients. CAZyme expression markedly differ during the infection of different hosts^28^ and some are even specifically up- and down-regulated. GH families (e.g., GH3, GH5, GH7, GH9, and GH17) play important roles in the fungal degradation of celluloses with different enzymatic activity, primarily β-1,3-1,4-glucanase, β-1,4-glucosidase, β-1,3 glucosidase, β-1,4-endoglucanas and cellobiohydrolase in this study. While GH families, such as GH15, GH16, GH35, GH51 and GH95, are involved in hemicellulose degradation through the activity of various enzymes, mainly α-fucosidase, α-glucuronidase and α-arabinofuranosidase as well as β-1,4-galactosidase and GH18, which are involved in chitin degradation, GH13, GH15 and GH18 families are involved in starch degradation^90^. Interestingly, the GH13 member a-amylase catalyses the hydrolysis of starch and sucrose in crops and has been previously studied^28^ but genes in the GH13 family, such as *AG1IA_09176*, *AG1IA_06735* and *AG1IA_00910*, were only specifically up-regulated in R-R in this study. In addition, *AG1IA_01811*, *AG1IA_04015* and *AG1IA_09356* belong to families, such as GH28, GH43 and PL1, which play a role in pectin breakdown and were highly and specifically expressed in S-S compared to S-R, in agreement with the fact that the cell walls of dicots contain higher levels of pectin than monocots^91^. The cell wall composition of dicots and monocots differs, particularly in terms of the proportion of pectin and hemicelluloses, and CAZyme family diversity is correlated with host specificity^92, 93^. Thus, our data are consistent with the hypothesis that pathogens of monocots are better adapted for degradation of monocot cell walls while pathogens of dicots are better adapted for degradation of dicot cell walls, reflecting host preferences^90^. To a certain extent, strains of *R. solani* AG1IA can adapt to different hosts and use different mechanisms of infection, depending on the host. These important findings will lay the groundwork for studies of the pathogen-host interaction of *R. solani* and the discovery of a large number of DEGs will enable further studies of the intrinsic differences in the pathogenicity in *R. solani* AG1IA towards different hosts. In turn, this may lead to the development of molecular breeding strategies for selecting species-specific resistant breeding materials in different hosts.

## Materials and Methods

### Origin, isolation and identification of strains

Three sequenced strains of *R. solani* AG1IA were used in this study. The three strains, rice IA, maize IA, and soybean IA, were randomly isolated from heavily infected rice (*O. sativa L*.), maize (*Z. mays L*.) and soybean (*G. max*) plants in Sichuan province in areas different altitudes (Xichang, Chengdu, and Yaan). The infected samples were thoroughly washed with sterile water, cut into small pieces, surface-sterilized with 1% sodium hypochlorite solution, and plated on agar containing no added nutrients^94^. Target strains were obtained by hyphae tip transplantation, and the purified strains were transferred to a block of PDA (15 g/L, Amresco, Solon, OH, USA) cultured at 28 °C for 24 h and stored at 4 °C. The three ecotypes showed identical ITS1 and ITS4 rDNA sequences, microscopic appearances, and abilities to cause disease in maize and soybean cultivars^95^.

### Anastomosis test

An anastomosis test was performed according to the method of S. Stepniewska-Jarosz^96^. Cultures of three isolated *R. solani* stains were paired with known *R. solani* AG1IA obtained from the Erxun Zhou, South China Agricultural University. The comparisons were performed on microscope slides with a sterile piece of cellophane predipped in a PDA agar medium before use. Three 5-mm disks from young *R. solani* cultures were placed on the slide (the tester in the centre and two unidentified *R. solani* isolates). The slides were placed in a moist chamber and incubated in the dark at 28 °C for 18–20 h until the hyphae from opposite disks overlapped. The overlapping portion was examined under the microscope for hyphal fusion observation.

### Growth and mycelia

Small pieces of mycelium were removed from the margin of the mycelia and added to fresh solid PDA at 28 °C for 1 week. This was repeated three times. The mycelium diameter was measured every 12 h to assess growth, and changes in mycelia colour were observed.

### Disease index test

The detached leaf method was used to assess the pathogenicity of rice IA, maize IA, and soybean IA^97^. Samples of cultivar 93–11 (rice), ZhengHong 505 (maize) and NanDou 5 (soybean) of similar sizes and leaf ages were placed on moist filter paper in a porcelain dish after being washed in sterile water. A block of PDA containing 2-day-old mycelia from *R. solani* AG1IA was placed in the middle of the leaf surface, covered with plastic wrap, and placed at 28 °C with 78% humidity. The leaves were observed every 12 h from 0–48 h post infection and speckles of disease were counted. Control leaves were inoculated with fresh PDA. Each experiment was repeated in triplicate. ImageJ software (NIH, Bethesda, MD, USA) was used to determine the spot area^98^.

### Sample treatment, total RNA extraction, cDNA library construction, and sequencing

Rice leaves inoculated with rice IA (R-R), maize IA (M-R) and soybean IA (S-R), maize leaves inoculated with maize IA (M-M), and soybean leaves inoculated with soybean IA (S-S) were cultured in individual petri dishes at 28 °C with 78% humidity. Rice IA (R0), maize IA (M0), and soybean IA (S0) were transferred to PDA at the same time and under the same culture conditions as in the experiment group. When more than half of the leaves exhibited lesions, mycelia were removed from the leaves by scraping^28^ and frozen in liquid N_2_. Total RNA was extracted using a Fungal RNA Kit (Omega, Norcross, GA, USA), following the manufacturer’s recommendations, and suspended in DEPC-treated water. RNA quantity was assessed with a NanoDrop2000 spectrophotometer (Thermo Scientific, Waltham, MA, USA). After all of the samples were confirmed by sequencing, they were sent to Beijing Biomarker Technologies (Beijing, China) for cDNA library construction and sequencing. Libraries were prepared using NEBNext mRNA Library Prep Master Mix Set for Illumina (NEB, E6110) and NEBNext Multiplex Oligos for Illumina (NEB, E7500) according to manufacturer’s instruction and paired-end sequencing was done using Illumina Genome Analyzer II technology (Illumina) to obtain reads of 100 bp length. Original paired-end reads were identified, and low-quality and primer/adapter-contaminated reads were filtered using NGSQC Toolkit^99^. The high quality reads were used for downstream analysis after quality filtering. Then, transcripts were aligned to scaffold genome sequences using BLAST^100^. The sequencing data generated in this study have been deposited in Sequence Read Archive (SRA) data base and under the accession number SRP106262.

### Alternative splicing analysis and validation

Alternative splicing events in *R. solani* AG1IA genes were identified using transcriptome sequences from the published *R. solani* AG1IA genome^29^. The alternative splicing events were constructed by assembling transcripts of the genome (Genome-guided Trinity for Gene Structure Annotation, http://trinityrnaseq.sourceforge.net/genome_guided_trinity.html). RNA-seq reads were aligned to the genome using GSNAP^101^. The genome-based transcripts were assembled using Trinity^102^. With GMAP and PASA^103^, AS events in genes were identified. RT-PCR was used for alternative splicing validation and cDNA was used as a template to perform RT-PCR. Primers of *AG1IA_03552* are listed (Supplementary Table S10).

### Gene ontology enrichment and pathway analysis

The GO terms or metabolic pathways significantly enriched in DEGs were identified by performing functional enrichment analyses. The Nr BLLAST results were imported into the Blast2 GO software for annotation with GO terms. This analysis involved mapping of all of annotated genes to GO terms in the database and enumeration of the genes associated with each term. Then a Perl script was used to plot the GO functional classifications for unigenes with a GO term hit to visualize the distribution of gene functions. The obtained annotation was enriched and refined using Top GO (R package) with the “elim” method and Kolmogorov-Smirnov test. GO terms with a corrected *q* value of <0.05 were considered significantly enriched in DEGs.

### Differential expression analysis

In this study, gene expression (false discovery rate [FDR ≤ 0.01) was assessed using EdgeR^104^ software. The FDR was used as a key indicator for screening significantly differentially expressed genes. The transcripts with log_2_ fold change ≥1 (up-regulated genes) and ≤(−1) (down-regulated genes) with FDR cut off ≤0.05 were considered as significantly differentially expressed genes.

### Prediction, annotation, and analysis of secreted proteins

Secretomes were predicted following two rules: the gene contains a signal peptide at the N-terminus, as predicted by at least two types of software, and the gene lacks a transmembrane helix as predicted by at least one type of software. Prediction software included SignalP 4.0 (http://www.cbs.dtu.dk/services/SignalP)^105^, TargetP 1.1 (http://www.cbs.dtu.dk/services/TargetP)^106^, Phobius (http://phobius.sbc.su.se), PrediSi (http://www.predisi.de/index.html) and TMHMM 2.0 (http://www.cbs.dtu.dk/services/TMHMM). Extracellular proteins were predicted using Wolfpsort^107^. Secreted proteins with a maximum of 200 amino acids and at least 4% cysteine residues were considered cysteine-rich proteins. Effector P (http:// effectorp.csiro.au/) was also used to predicted genes which show probability for being secreted effectors. Multi Experiment Viewer_ENREF_110 software was used for making heat map.

### Candidate gene expression analysis via qRT-PCR

A total of twelve candidate genes belonging to different categories from RNA-seq were used for gene expression analysis via qPCR. First-strand cDNA was synthesized from total RNA using a Transcript First Strand cDNA Synthesis Kit (Roche, Upper Bavaria Germany). qPCR was performed using the SoAdvanced Universal SYBR Green Supermix (Bio-Rad, Hercules CA, USA). The qPCR reactions were performed in a final volume of 10 μL containing 5 μL of 2 × MasterMix, 0.5 μL of 100 μM of each primer, 1 μL of ddH_2_0 and 3 μL of cDNA. The reactions occurred at 95 °C for 30 sec, followed by 40 cycles of 95 °C for 10 sec, 58 °C for 30 sec, and melting curve analysis from 65 °C to 95 °C at 0.5 °C increments. Primers for qPCR were designed based on our predicted gene sequences (Supplementary Table S11). Primers for 18 S were used for internal controls, and all of the qPCR primers were tested with RT-PCR before use. Fold changes were determined by the 2^−ΔΔCt^ method based on three technical replicates per sample. All qPCR reactions were repeated at least three times.

## Electronic supplementary material

Supplementary information
