# Supplementary material for: Transcriptome analysis reveals the host selection fitness mechanisms of the Rhizoctonia solani AG1IA pathogen
Source: Sci Rep. 2017 Aug 31;7:10120. doi: 10.1038/s41598-017-10804-1 (PMC5579035; doi:10.1038/s41598-017-10804-1)
Supplement: Supplementary file 1 — Supplementary information [file 41598_2017_10804_MOESM1_ESM.pdf]

Transcriptome analysis reveals the host selection fitness mechanisms of the *Rhizoctonia solani* AG1IA pathogen

Yuan Xia[1], Binghong Fei[1], Jiayu He[1], Menglin Zhou[1], Danhua Zhang[1], Linxiu Pan[1], Shuangcheng Li[1,3], Yueyang Liang[1,3], Lingxia Wang[1,3], Jianqing Zhu[1,3], Ping Li[1,3] and Aiping Zheng[1,2]

[1]Rice Research Institute of Sichuan Agricultural University, Chengdu 611130, China, [2]Key Laboratory of Sichuan Crop Major Diseases, Sichuan Agricultural University, Chengdu 611130, China. [3]Key Laboratory of Southwest Corp Gene Resource and Genetic Improvement of Ministry of Education, Sichuan Agricultural University, Ya'an, 625014, China.

Author for correspondence: apzh0602@gmail.com

Supplementary tables

Samples	Total reads	Total base(bp)	GC%	Q20	Q30
R0	14,714,110	3,704,361,594	52.88	92.42	92.42
R-R	11,702,595	2,946,184,392	51.72	91.77	86.81
M-M	12,366,055	3,113,631,011	52.63	92.31	87.41
M0	13,479,284	3,393,042,636	52.4	91.58	86.34
M-R	13,174,572	3,316,479,415	52.66	92.03	86.95
S-S	14,144,703	3,560,675,097	51.64	91.73	86.43
S0	13,293,517	3,344,583,075	52.38	91.86	86.82
S-R	12,096,744	3,044,104,095	52.66	92.26	87.35

Table S1. Original sequencing data evaluation.

Statistical content	Statistical content	Total reads	Mapped reads	Unique mapped reads	Multiple mapped reads	Pair mapped reads	Single mapped reads
R0	Number	29,428,220	23,338,124	23,072,089	266,035	20,677,317	2,467,739
	Percentage	100%	79.31%	98.86%	1.14%	88.60%	10.57%
R-R	Number	23,405,190	18,354,846	18,088,818	266,028	16,195,604	1,996,940
	Percentage	100%	78.42%	98.55%	1.45%	88.24%	10.88%
M-M	Number	24732110	18383840	18196151	187689	16020736	2242824
	Percentage	100%	74.33%	98.98%	1.02%	87.15%	12.20%
M0	Number	26958568	19754216	19557018	197198	17179302	2426538
	Percentage	100%	73.28%	99.00%	1.00%	86.97%	12.28%
M-R	Number	26349144	19456535	19229635	226900	17019263	2315409

	Percentage	100%	73.84%	98.83%	1.17%	87.47%	11.90%
S-S	Number	28289406	19594330	19303935	290395	16733805	2715249
	Percentage	100%	69.26%	98.52%	1.48%	85.40%	13.86%
S0	Number	26587034	18329513	18145815	183698	15580823	2621432
	Percentage	100%	68.94%	99.00%	1.00%	85.00%	14.30%
S-R	Number	24193488	17013843	16775483	238360	14522443	2377085
	Percentage	100%	70.32%	98.60%	1.40%	85.36%	13.97%

Table S2. Transcriptome reads mapped to *Rhizoctonia solani* AG1 IA genome

Annotation Database	Annotated_Number	300<=length<1000	length>=1000
COG_Annotation	496	178	312
GO_Annotation	495	184	300
KEGG_Annotation	230	75	123
Swissprot_Annotation	764	270	485
nr_Annotation	1565	630	906
All_Annotated	1565	630	906

Table S3. Numbers of functional annotation of novel genes

Sample	R-R	R0	M-M	M0	M-R	S-S	S0	S-R

Alt3' splice site	2159	1951	2050	2020	2074	1939	2075	1998
Alt5' splice site	1933	1813	1819	1846	1837	1789	1840	1813
AltFirstExon	392	373	385	360	388	401	364	390
AltLastExon	369	306	362	366	357	360	411	366
IntronRetention	4517	4782	4417	4474	4556	4331	4459	4357
skippedExon	1971	1805	1838	1920	1914	1799	1871	1870
total_AS events	11341	11030	10871	10986	11126	10619	11020	10794

Table S4. Number of each type of alternative splicing events in eight transcriptomes

Sample	R-R	R0	M-M	M0	M-R	S-S	S0	S-R
Alt3' splice site	301	277	285	307	263	249	281	260
Alt5' splice site	282	247	250	253	254	243	259	247
AltFirstExon	45	54	46	50	56	49	60	51
AltLastExon	63	48	56	51	49	54	71	63
IntronRetention	577	656	557	607	571	544	571	549
skippedExon	250	236	224	257	244	220	236	256
total_AS genes	1518	1518	1418	1525	1437	1359	1478	1426
proportion	17.42%	17.36%	16.02%	17.18%	16.10%	15.10%	16.53%	15.60%

Table S5. Comparison of AS distribution in predicted critical pathogenesis-associated genes. The proportion indicates the percentage of critical pathogenesis-associated alternative spliced genes during different host infection.

Gene ID	Sample	Nr_annotation	AS type
	R-R		Alt5' site
AG1IA_04402	M-M	2-deoxy-D-gluconate 3-dehydrogenase	Alt5' site
	M-R		Alt5' site

	S-S		AltFirstExon	
	S-R		Alt5' site	
	R-R		Alt5' site<br>IntronRetention	Alt5' site<br>skippedExon
AG1IA_00285	M-M	cytochrome P450 domain-containing protein	Alt5' site<br>skippedExon	Alt5' site
	M-R		IntronRetention	skippedExon
	S-S		skippedExon	
	S-R		skippedExon	
	R-R		IntronRetention	
	M-M		IntronRetention	
AG1IA_09291	M-R	glycoside hydrolase family 16 protein	IntronRetention	
	S-S		IntronRetention	
	S-R		IntronRetention	
	R-R		Alt5' site	
	M-M		Alt5' site	
AG1IA_09535	M-R	efhand domain-containing protein	Alt5' site	skippedExon
	S-S		Alt5' site	
	S-R		Alt5' site	skippedExon
	R-R		IntronRetention	Alt3' site
	M-M		IntronRetention	Alt3' site
AG1IA_04262	M-R	MFS_1 domain-containing protein	IntronRetention	Alt3' site
	S-S		IntronRetention	Alt3' site
	S-R		IntronRetention	
AG1IA_01513	R-R	hypothetical protein	IntronRetention	
	M-M		IntronRetention	

	M-R		IntronRetention	
	S-S		IntronRetention	
	S-R		IntronRetention	
	R-R		IntronRetention	
	M-M		IntronRetention	
AG1IA_06281	M-R	IncA domain-containing protein	IntronRetention	
	S-S		IntronRetention	
	S-R		IntronRetention	
	R-R		IntronRetention<br>skippedExon	Alt3' site
	M-M		IntronRetention<br>skippedExon	Alt3' site
AG1IA_00042	M-R	sugar (and other) transporter domain-containing protein	IntronRetention<br>skippedExon<br>AltLastExon	AltFirstExon<br>Alt3' site
	S-S		IntronRetention	skippedExon
	S-R		IntronRetention<br>AltLastExon	Alt3' site<br>skippedExon

Table S6. Commonly alternative spliced genes under all host infection conditions.

Gene ID	R0 FPKM	R-R FPKM	M0 FPKM	M-R FPKM	M-M FPKM	S0 FPKM	S-R FPKM	S-S FPKM
AG1IA_00058	14.6511	7.27246	6.62856	3.89001	2.79449	25.0217	2.84238	3.10135
AG1IA_00157	153.2838	92.4913	22.995937	23.68	24.10202	124.7511	43.764	63.1699
AG1IA_00192	32.60532	36.32015	34.39902	40.0529	30.81672	39.13962	38.56928	99.6494
AG1IA_00273	0.975175	26.7059	8.70984	18.0522	21.1686	22.741	9.31206	68.3488

AG1IA_00396	55.8657	16.27326	26.314545	16.161258	23.23721	23.262473	14.047	22.81639
AG1IA_00550	31.2993	20.2479	13.4835	16.4411	21.4966	20.9484	22.4789	75.7371
AG1IA_00669	530.66628	325.55722	200.191	187.916	282.94709	282.73619	140.45324	81.799969
AG1IA_00748	20.7749643	17.224763	24.858411	16.722081	20.7908434	14.703211	7.342422	9.865729
AG1IA_00879	228.3126	196.20316	161.3733489	142.4623	147.23119	132.8096	104.24613	56.97752
AG1IA_00925	223.259621	1314.55997	757.968283	1014.672497	1265.229236	710.19516	1600.83864	1173.45717
AG1IA_01042	0.383168	20.305173	4.73197	6.6521	13.35267	17.97448	22.75454	115.87259
AG1IA_01137	308.0938	542.228	633.377	494.838	1270.814	496.327	1284.583	903.375
AG1IA_01512	41.8685	27.81397	31.2917	25.21925	29.98825	20.82541	40.448	46.3435
AG1IA_01604	70.4311	256.783	178.641	125.167	192.844	326.466183	193.145	256.012718
AG1IA_01632	8.04841	6.98022	15.60996	27.39766	14.76294	15.94215	7.00799	9.46533
AG1IA_01647	8.31628	17.0302	15.6174	16.4019	20.8763	18.5137	20.491	22.111
AG1IA_01812	17.5474	8.82254	16.8445	16.6231	16.0928	10.91587	43.1211	86.9936
AG1IA_02024	11.7822	6.56882	8.72049	8.40775	9.05104	4.32121	19.421	10.5321
AG1IA_02138	65.02996	120.59864	111.62953	145.6184	152.5003	137.84613	163.78005	66.90129
AG1IA_02188	36.0703	36.375	30.714	29.2736	26.8847	33.8135	37.7017	77.4341
AG1IA_02358	27.1721	5.35594	7.02617	4.37648	5.3791	6.62649	5.39134	6.59631
AG1IA_02386	8.077	2.82103	5.73554	4.12429	4.70911	1.75989	4.14676	2.6197
AG1IA_02874	88.8716	38.9206	20.599715	18.80501	15.7787	25.03009	27.28923	76.1832
AG1IA_02928	37.02895	18.99869	33.91192	8.781994	12.909439	33.61054654	6.12291275	12.24763712
AG1IA_02940	5.740342	10.352061	141.079684	24.15545	20.24686	18.481737	25.375485	5.569006
AG1IA_03245	4.28978	8.99907	8.44859	6.99196	8.64658	12.9581	9.20884	6.59426
AG1IA_03763	1.960376	1.5384	0.946286	0.619728	0.917254	0.834423	2.05219	3.47325
AG1IA_03834	85.67403089	99.41817	90.413895	116.5071	115.22809	107.119194	100.17772	314.80959

AG1IA_04006	669.722	175.497	44.7738	87.4342	38.9683	85.2841	70.1771	132.825
AG1IA_04144	735.12873	339.89873	475.5496	255.68898	215.2577	313.4746121	222.75856	82.37355438
AG1IA_04237	25.8462	3.36074	14.80215	3.036583	3.82669	6.92131	1.606893	14.00578
AG1IA_04497	6.63837	8.98644	10.4354	10.9383	18.7853	9.58512	11.3816	43.6471
AG1IA_04714	23.718	37.0131	25.0757	43.5326	37.9557	60.2832	29.3458	86.9279
AG1IA_04889	56.8993	48.85625	95.5903	50.71931	43.22839	78.2033	22.92902	17.14699
AG1IA_04923	3.07341	4.39288	1.76134	2.67074	1.85283	9.32985	3.59326	2.5948
AG1IA_04998	64.1409	178.00587	220.63455	246.76624	269.32505	132.81132	278.42823	335.44632
AG1IA_05179	1.5338	0.860656	1.65766	0.753362	0.367774	0.675542	6.04582	2.18101
AG1IA_05291	29.011	36.5826	33.8192	112.545	50.6306	120.723	126.412	72.316
AG1IA_05454	4221.182	1331.7781	1596.698	2096.811	2639.125	2403.823	2259.191	4266.164
AG1IA_05500	895.99	1477.814	477.736	874.498	935.689	414.777	430.397	185.5044
AG1IA_05506	25.918	6.40453	9.324487	12.56	8.6646	9.26979	13.0412	27.3191
AG1IA_05880	64.25	24.6955	25.0875	27.9003	46.9424	112.908854	75.31191	24.5879
AG1IA_05955	16.9079	65.6027	43.0483	56.3306	37.7554	73.2405	41.4316	30.264
AG1IA_06272	53.99179	252.5603	166.92256	218.04269	227.26189	198.3696601	250.18288	264.6091519
AG1IA_06325	868.57705	922.3337148	694.9023	1027.2429	833.482	1294.2517	1317.604	2782.853
AG1IA_06385	761.4265	795.82224	608.05112	619.82165	294.06928	802.302153	428.2674	264.16141
AG1IA_06394	12.6275	60.8778	25.7054	53.7219	82.2022	83.004	53.6139	42.3979
AG1IA_06428	2.65653	1.11427	3.10306	1.56705	1.27738	2.285664	2.48251	4.27235
AG1IA_06613	1834.24191	3928.959	2640.69	2885.857	4236.7732	4604.26	5394.319	3148.55
AG1IA_06668	50.8977	4.39969	0	4.24686	3.65772	0	0.349262	0
AG1IA_06710	1.70773	0.836859	0	0	0	3.57972	1.07029	5.00146
AG1IA_06725	32.2231	36.0311	11.75254466	13.4785	12.747	30.743393	11.2523	19.80304

AG1IA_06741	2.43938	3.9082	5.95084	3.8718	5.17412	1.9805	1.45979	8.75349
AG1IA_06958	59.312684	5.676357	29.07027	5.844851	9.301868	26.77631	42.349847	9.5887
AG1IA_07075	81.95943	75.64224	24.72412	31.23134	57.21704	19.481	12.3105	69.06348
AG1IA_07262	1.60914	1.46884	0.944937	0.078609	1.04245	0.401205	1.14167	4.02148
AG1IA_07267	4.92714131	12.00052	7.594365868	8.773099933	10.538	13.39043683	9.64843	6.11922409
AG1IA_07280	212.8462	731.657	403.095	621.781	694.299	639.543	506.476	306.202
AG1IA_07307	0	0	4.25074	3.86958	0	10.1072	8.72118	80.875
AG1IA_07357	10.5112	3.37805	0	1.91249	0.00128761	1.19312	13.4289	18.2585
AG1IA_07490	45.143887	14.36346	19.143671	16.620335	16.46599	20.21557	23.944156	27.326866
AG1IA_07563	71.128873	36.7288	49.4138	23.50333	16.132633	193.9792	36.6503	50.163
AG1IA_07627	222.349	242.344	297.008	264.12	245.273	287.6367916	254.942389	774.252
AG1IA_07806	17.2402	18.4556	27.3742	29.4478	67.9142	26.5383	57.3327	34.6605
AG1IA_07973	550.8287	1901.51301	224.23868	1203.606	725.929	104.17961	436.281145	213.50576
AG1IA_07977	220.47399	275.4717	123.57722	293.4569	229.7352	143.8183	209.4137	211.3687
AG1IA_07978	60.416404	151.84285	19.5052	69.05649	39.481353	34.476962	121.725963	36.4046
AG1IA_08059	400.3862922	294.5575838	281.74058	308.5501588	229.2609016	238.7085412	401.080338	511.5099399
AG1IA_08303	36.2532	170.0447	0.2723143	1.657252	0.146395	214.4635	540.809	532.687
AG1IA_08310	18.7408	7.745956	18.213424	6.9873	8.731188	8.34489	10.202587	5.39085
AG1IA_08405	32.74519222	82.54377736	40.45593417	85.3663	62.32241	34.40905429	70.183174	92.125941
AG1IA_08487	43.39285	60.967202	34.6198	36.263	63.5405	31.8083	9.75183	59.4161
AG1IA_08488	15.86626	27.908505	30.311843	23.956699	25.005129	31.846085	37.401961	80.901562
AG1IA_08493	7.29984	6.64562	21.2564	6.69727	10.9398	14.3596	49.4868	39.3804
AG1IA_08580	18.11097	17.60219	22.38937	33.0509	23.61427	24.25921	25.78557	10.06844
AG1IA_08615	35.6833	30.16053	11.90492	11.709891	7.38434	8.3839088	19.58727	6.528516

AG1IA_08650	55.1698	48.6992	17.1278	9.02387	14.728	388.145	16.5117	7.32963
AG1IA_08777	6.45391	1.032367	3.8635	2.219544	3.172254	64.6132	1.8998	3.51675
AG1IA_08852	8.24112037	4.95346263	4.281598	15.573323	5.25308102	7.616356823	6.798016544	8.539497
AG1IA_08891	0.229257	0.160186	0	0.186623	0.206541	0	0.210698	1.55635
AG1IA_08892	2.34723	0	0.190372	0.191027	0.211484	0	0.215673	1.79223
AG1IA_08957	4.08127	2.91334	3.88914	0.815692	3.64053	3.88768	1.64742	4.81634
AG1IA_09055	6.66965	2.1848	3.87775	9.49395	6.9443	19.38819	31.2068	16.21232
AG1IA_09060	8.12502	9.52329	10.75147	18.59956	73.66833	24.03521	19.21846	17.418943
AG1IA_09202	57.8183	195.658	77.23661	221.03286	249.37167	280.95652	666.5236	565.28884
AG1IA_09203	68.7073	128.574	49.854487	184.786	113.749	99.9421	164.353	68.3009
AG1IA_09207	78.9049	1061.67	134.895	656.198	413.791	65.0901	527.969	548.323
AG1IA_09217	8.95407	18.7201	15.3597	22.8324	19.1861	19.4493	15.5632	18.4684
AG1IA_09303	6.892583659	8.70625	2.74558	5.341905866	10.61404	2.169812	3.02017405	3.7805943
AG1IA_09544	0	28.25532	0	0	0	0	0	0
AG1IA_09836	319.83227	169.5706453	261.40992	241.818809	327.31686	115.5117668	358.5839	813.4285
AG1IA_09837	428.28	186.699	259.407	257.077731	224.027	186.27908	291.644	257.574848
AG1IA_09956	46.2903	52.2652	98.9031	187.4	267.915	0	0	0
AG1IA_10043	2917.612	121.6334	395.2834	11.8818	5.239245	8561.17	227.0672	93.41286
AG1IA_10158	25.5207	21.3174	40.1037	27.1382	31.2311	15.8843	36.0448	35.5189
AG1IA_10180	0.986406	1.83241	1.8675	0	0.89002	1.08025	0.601919	0
AG1IA_10204	14.21	5.48856	6.40732	2.5519	5.70417	0.323694	2.16198	6.31978
AG1IA_10318	1.53899	5.33092	1.66632	5.36161	9.74768	0	0	0
AG1IA_10394	10.0522	26.8013	26.6842	21.9395	27.3525	20.2004	15.1247	13.3976
AG1IA_10403	219.775	84.34814	265.55654	111.88446	261.82721	353.7865	106.22048	535.6554

AG1IA_10405	43.2796	65.7565	75.46626	82.50115	62.85795	57.2981	189.75673	173.655
AG1IA_10423	23.9792	45.1646	43.7077	29.5254	38.1957	27.9981	24.5949	9.72641
AG1IA_10444	33.5946	107.266184	41.2555	54.20202	50.5987	35.22926	58.21992	29.11024
AG1IA_10485	71.396	41.9779	10.5627	16.1471	35.3266	34.0072	25.7471	14.4177

Table S7. FPKM of differentially expressed candidate effectors under all host infection conditions.

Gene ID	Nr_annotation
**AG1IA_01137**	hypoxia induced protein conserved region domain-containing protein
AG1IA_00157	polysaccharide deacetylase domain-containing protein
**AG1IA_00192**	peptidase s24-like domain-containing protein
**AG1IA_02188**	MRP-S33 domain-containing protein
**AG1IA_02874**	ribosomal l36 domain-containing protein
**AG1IA_03245**	kinase domain-containing protein
**AG1IA_03834**	LSM domain-containing protein
AG1IA_04714	Smr domain-containing protein
AG1IA_05454	B12D domain-containing protein
**AG1IA_06325**	FKBP-type peptidyl-prolyl cis-trans isomerase domain-containing protein
AG1IA_06385	extracellular elastinolytic metalloproteinase
AG1IA_00669	glycosyl hydrolase family 61 domain-containing protein
**AG1IA_07075**	CFEM domain-containing protein
**AG1IA_07280**	HSP70 domain-containing protein
AG1IA_07490	Yippee domain-containing protein
AG1IA_07563	erv1/alr family domain-containing protein
**AG1IA_07627**	zf-rbx1 domain-containing protein

**AG1IA_08303**	peroxidase, family 2 domain-containing protein
AG1IA_08310	DDHD domain-containing protein
**AG1IA_08405**	coenzyme A transferase domain-containing protein
AG1IA_08487	Ribonuclease domain-containing protein
**AG1IA_08488**	Ribonuclease domain-containing protein
AG1IA_08777	plastocyanin-like domain-containing protein
**AG1IA_09055**	thaumatin-like protein
**AG1IA_09060**	thaumatin-like protein
**AG1IA_00925**	Barwin domain-containing protein
**AG1IA_09303**	subtilase family domain-containing protein

Table S8. Annotation of different expressed genes in candidate effectors. The positive training data set (experimentally verified fungal effectors) that was used for training EffectorP can be found in http://effectorp.csiro.au/data.html. A total of 27 genes out of 104 candidate effectors with a predicted function have been listed in table and genes which were bolded indicates up-regulated genes during host infection.

Gene ID	Family	Enzyme activity	Substrates
AG1IA_00122	GH9	lichenase / endo-β-1,3-1,4-glucanase	cellulose
AG1IA_00256	GH3	β-1,4-glucosidase	cellulose
AG1IA_09735	GH17	β-1,3glucosidase	cellulose
AG1IA_05285	GH5	β-1,4-endoglucanas	cellulose
AG1IA_01218	GH3	β-1,4-glucosidase	cellulose
AG1IA_02923	GH5	β-1,4-endoglucanas	cellulose
AG1IA_06159	GH7	Cellobiohydrolase	cellulose
AG1IA_02003	GH3	β-1,4-glucosidase	cellulose
AG1IA_08771	GH5	β-1,4-endoglucanas	cellulose

AG1IA_05561	GH17	β-1,3glucosidase	cellulose
AG1IA_07901	GH95	α-fucosidase	hemicellulose
AG1IA_06464	GH115	α-glucuronidase	hemicellulose
AG1IA_06014	GH51	α-arabinofuranosidase	hemicellulose
AG1IA_05653	GH51	α-arabinofuranosidase	hemicellulose
AG1IA_09291	GH16	β-1,4-galactosidase	hemicellulose
AG1IA_01054	GH35	β-1,4-galactosidase	hemicellulose
AG1IA_01811	GH28	Endo-/exo-(rhamno)galacturonases	pectin
AG1IA_04015	GH43	β-1,4-xylosidase	pectin
AG1IA_09356	PL1	Pectin lyase	pectin
AG1IA_02234	GH28	Endo-/exo-(rhamno)galacturonases	pectin
AG1IA_07255	GH18	chitinase	chitin
AG1IA_07247	GH18	chitinase	chitin
AG1IA_09176	GH13	α-amylase	Starch
AG1IA_06735	GH13	α-amylase	Starch
AG1IA_00910	GH13	α-amylase	Starch
AG1IA_04992	GH15	glucoamylase	Starch
AG1IA_02936	GH16	β-1,4-galactosidase	starch
AG1IA_08014	GH32	Inulinase	Inulin

Table S9. Putative differentially expressed up-regulated CAZyme genes involved in plant cell wall degradation of *R. solani* AG1IA based on the CAZy database (http://www.cazy.org).

Gene ID	Premier
AG1IA_03552 forward	5' GAACAGTCGAACAAAGTGGAGT 3'
AG1IA_03552 reverse	5' GCCGGAACAGTGTTGGTG3'

Table S10. Primers used in validation of AS.

Gene ID	Primer
AG1IA_07681 forward	5'GGAAGCCATCGAGGTCACAT 3'
AG1IA_07681 reverse	5'TGGTCGAAAGAGTTGACGCC 3'
AG1IA_07285 forward	5'GCGGACGGAAATGGACAC 3'
AG1IA_07285 reverse	5'CACCCAGTTGATACCTGAGATG 3'
AG1IA_01651 forward	5'CTGGCAATCTGCTACGGTCA3'
AG1IA_01651 reverse	5'TAGTGAAAGCGGCGCAAGAA3'
AG1IA_09202 forward	5'TTGCTCGCTTGCTCTGCC 3'
AG1IA_09202 reverse	5'CCGATTCACCAACAGTAGCC 3'
AG1IA_08167 forward	5'TCTTGCGGTCACAGTTCG 3'
AG1IA_08167 reverse	5'CGTCTTCGTCGTCCCATT 3'
AG1IA_06011 forward	5'ACTTCCATAGACGGTCAGC 3'
AG1IA_06011 reverse	5'CTACATTCCGAGGCGATT 3'
AG1IA_09369 forward	5'GACCGAGTAAACGCCAGTG 3'
AG1IA_09369 reverse	5'AGTATCCGGCTTCCATCC 3'
AG1IA_09664 forward	5'CTGCATGTGAGCGTTATGGC
AG1IA_09664 reverse	5'ATAGATGGCTTTCGGTGCGT

AG1IA_01129 forward	5'CTGGCTATGTTGGAACGGGT3'
AG1IA_01129 reverse	5'TTCGTCACGGTAAAGCACCA3'
AG1IA_05374forward	5'AAGGCGCAGAGAAGTTCGAG3'
AG1IA_05374 reverse	5'GACGACGCTGTATTTGGGGA3'
AG1IA_08104 forward	5'GCGGGGAACTATAGCTGGTC3'
AG1IA_08104 reverse	5'CTTCGGTGCCCATGCAGATA3'
AG1IA_01042 forward	5'TGCCTGCGGTTGTGCTATAA3'
AG1IA_01042 reverse	5'CCATCGCCTCGATTCCCAAT3'
18S forward	5'AATTCCAGCTCCAATAGCGT3'
18S reverse	5'TACATACCGTGAGGCAGACC3'

Table S11. RT-qPCR primers used in gene expression validation.

Supplementary figures

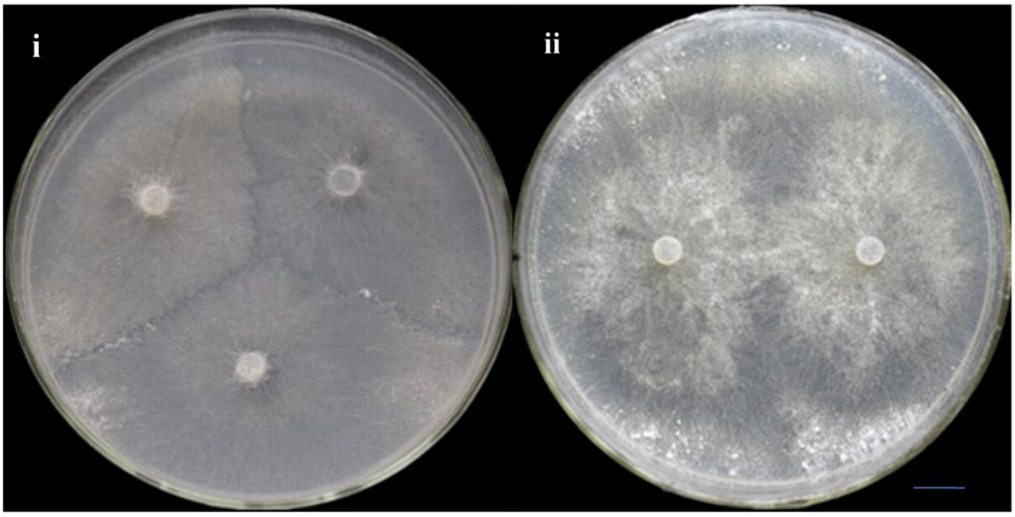

Figure S1. Somatic compatibility reactions of *R. solani* AG1 IA ecotypes. Somatic compatibility reactions of *R. solani* AG1 IA ecotypes on potato-dextrose agar (PDA). Scale bars, 2 cm. (i) Somatically incompatible intermediate interactions in rice IA, maize IA and soybean IA. (ii) Somatically compatible interactions in rice IA-rice IA.

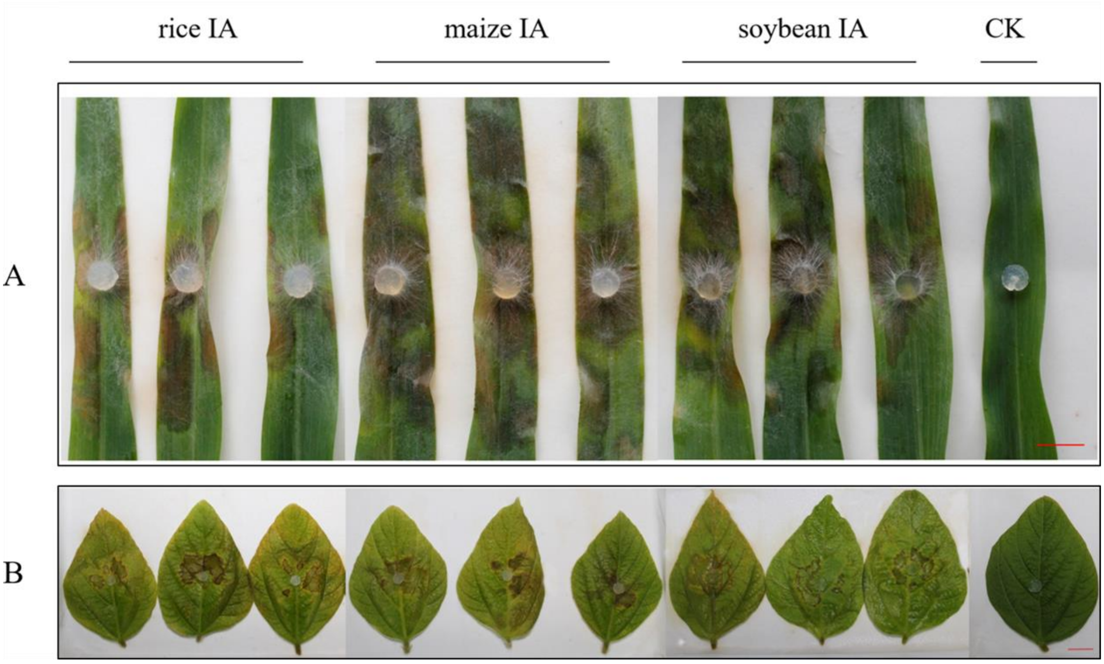

Figure S2 Pathogenic phenotype of rice IA, maize IA and soybean IA during the infection of maize and soybean. Area of spots caused by rice IA, maize IA and soybean IA at 48 hpi for maize (A) and soybean (B), respectively. Spot areas caused by maize IA were significantly more severe than spots caused by rice IA and soybean IA during infection of maize. Scale bars, 1 cm.

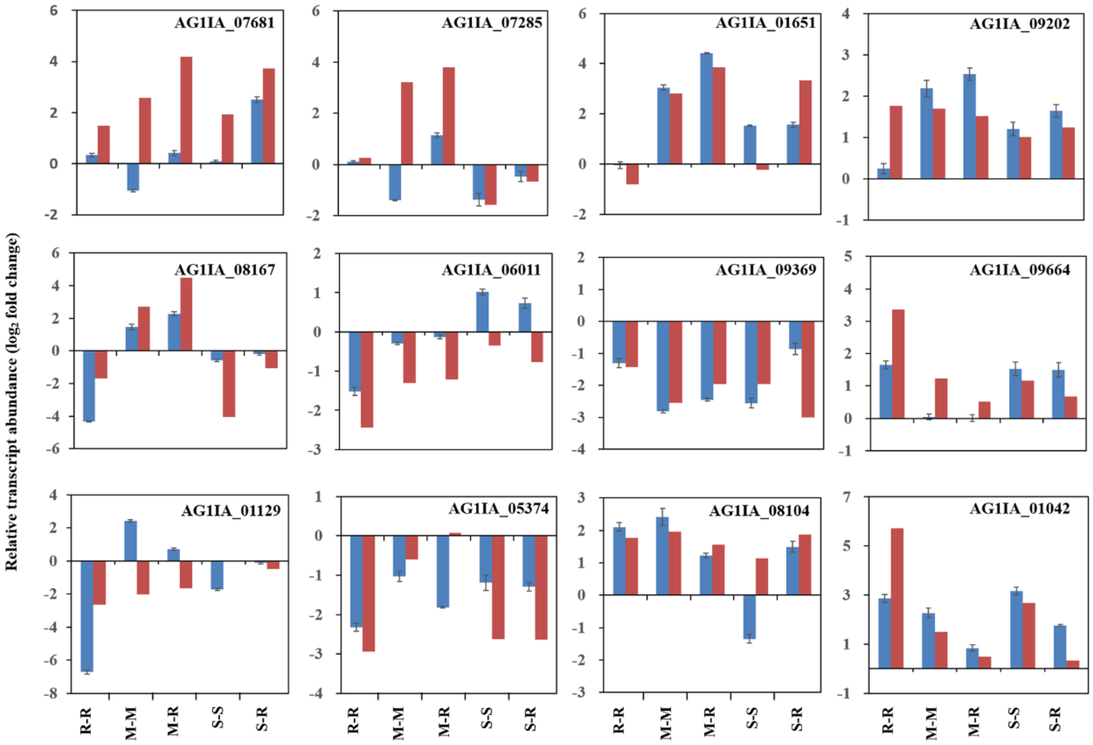

Figure S3. Validation of RNA-Seq data *via* qRT-PCR. Histogram depicting relative transcript abundance of selected transcripts in strains during different host infections. All data points are represented as log_2 fold change values. R-R was calculated using R0 as a reference, and expression under M-M, M-R, S-S and S-R was calculated with respect to control conditions for each cultivar (M0 and S0). Genes belonging to different categories, such as secreted proteins (*AG1IA_01042* and *AG1IA_01651*), candidate effectors (*AG1IA_07285* and *AG1IA_09664*), CAZymes (*AG1IA_01129* and *AG1IA_08104*) and cytochrome P450 (*AG1IA_08032* and *AG1IA_05374*), are included. The blue columns represent expression *via* qRT-PCR, and red columns represent the expression *via* RNA-Seq. Error bars, ± SE of the means of the raw data.

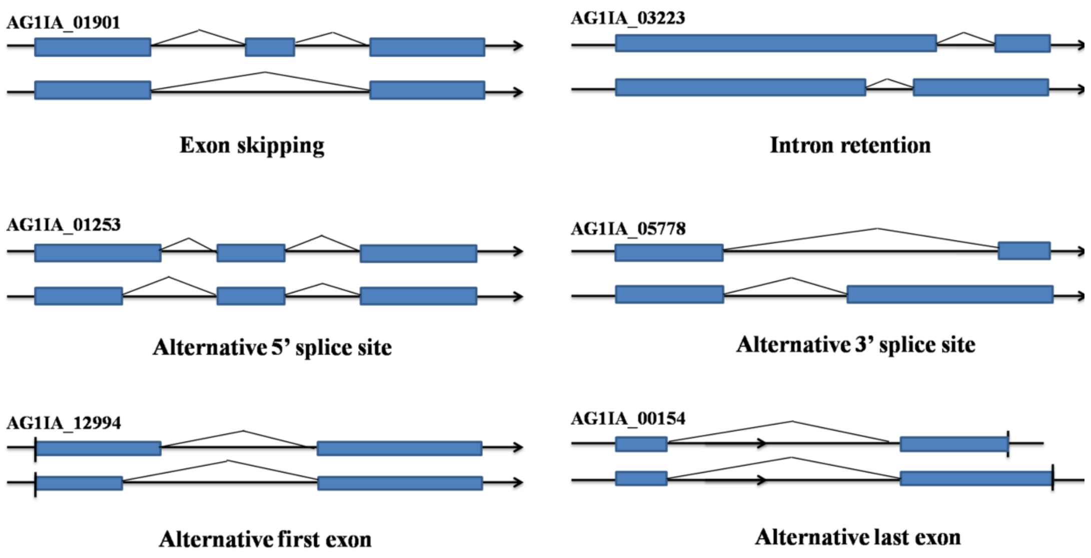

Figure S4. Predicted models of six types of alternative splicing events. Some predicted genes in M-M are listed, including *AG1IA_01901* (exon skipping), *AG1IA_03223* (intron retention), *AG1IA_01253* (alternative 5' splice site), *AG1IA_05778* ((alternative 3' splice site), *AG1IA_12994* (alternative first exon) and *AG1IA_00154* (alternative last exon).

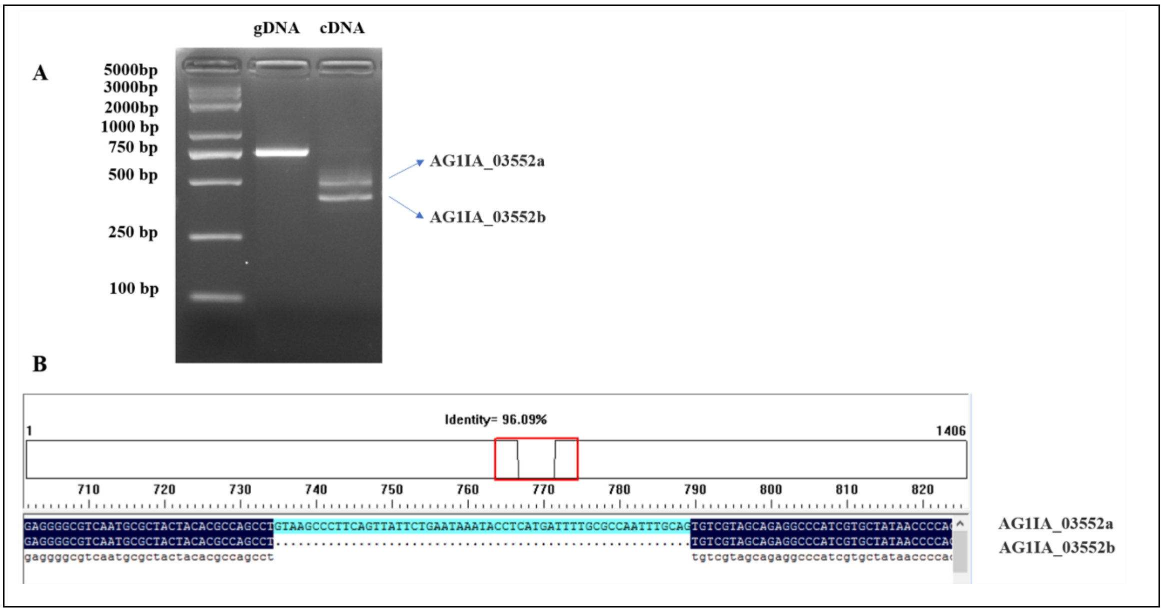

Figure S5. Identification of alternatively spliced genes. (A) *AG1IA_03552* with intron retention was selected for confirmation by RT-PCR in R-R. Genomic DNA template (middle lane) was used as a control. Primers were designed for flanking intronic regions. (B) DNAMAN was used for sequencing alignment. An intron is retained in *AG1IA_03552*a compared to *AG1IA_03552*b.

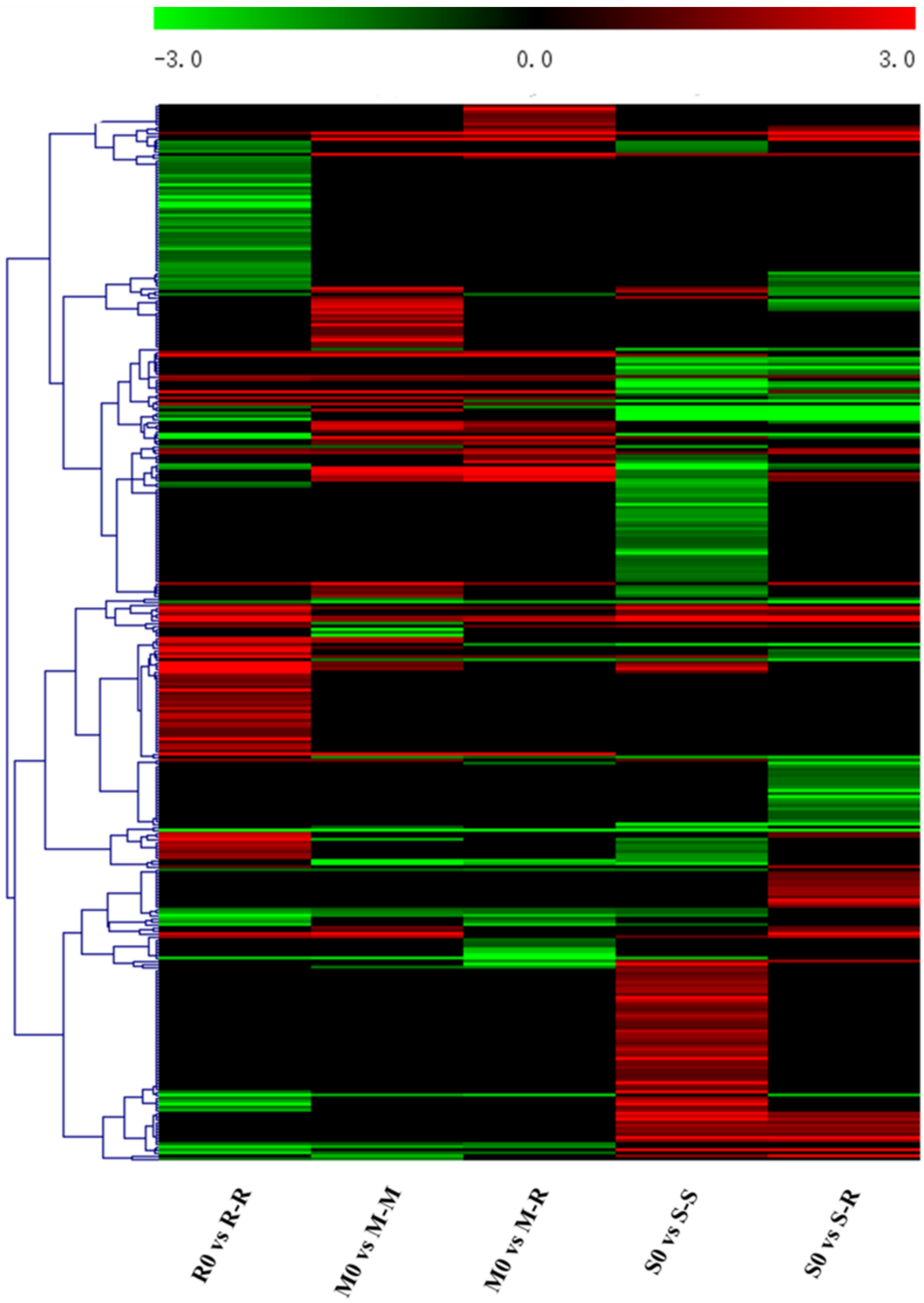

Figure S6. Differential expression of secreted proteins of *R. solani* AG1 IA during infection of different hosts. In total, 347 DEGs of secreted proteins were analysed by prediction algorithms; a hierarchical clustering statistic method based on Pearson's correlation (correlation≥98%) and average linkage was used. Red indicates a significant increase in gene expression, black indicates no change in expression and green indicates a significant decrease in gene expression (|log2FC|≥1). Colour scale represents $\log_2$ fold

change values from -3 to 3.
